# Decoupling epithelial-mesenchymal transitions from stromal profiles by integrative expression analysis

**DOI:** 10.1038/s41467-021-22800-1

**Published:** 2021-05-10

**Authors:** Michael Tyler, Itay Tirosh

**Affiliations:** Department of Molecular Cell Biology, Weizmann Institute of Science, Rehovot, Israel

**Keywords:** Cancer genomics, Cancer microenvironment, Data mining, Gene expression

## Abstract

Epithelial-to-mesenchymal transition (EMT) is the most commonly cited mechanism for cancer metastasis, but it is difficult to distinguish from profiles of normal stromal cells in the tumour microenvironment. In this study we use published single cell RNA-seq data to directly compare mesenchymal signatures from cancer and stromal cells. Informed by these comparisons, we developed a computational framework to decouple these two sources of mesenchymal expression profiles using bulk RNA-seq datasets. This deconvolution offers the opportunity to characterise EMT across hundreds of tumours and examine its association with metastasis and other clinical features. With this approach, we find three distinct patterns of EMT, associated with squamous, gynaecological and gastrointestinal cancer types. Surprisingly, in most cancer types, EMT patterns are not associated with increased chance of metastasis, suggesting that other steps in the metastatic cascade may represent the main bottleneck. This work provides a comprehensive evaluation of EMT profiles and their functional significance across hundreds of tumours while circumventing the confounding effect of stromal cells.

## Abstract (web summary)

Epithelial cancer cells can transition into a mesenchymal phenotype to enable invasion and metastasis. Here, the authors use previously published single-cell and bulk RNA sequencing datasets to decouple the mesenchymal expression profiles of cancer and stromal cells.

## Introduction

Metastasis is the main cause of death in cancer, but its underlying mechanisms remain unclear. The most commonly cited mechanism for metastasis in carcinoma is epithelial-to-mesenchymal transition (EMT), a process which occurs during normal development and wound healing and is thought to be co-opted by cancer cells^1,2^. In the classical form of this process, epithelial cells lose their cell-cell junctions and convert their rigid keratin cytoskeleton to a more flexible Vimentin-based structure. They gain the ability to modify and bind to elements of the extracellular matrix (ECM) by expressing proteins such as fibronectin, integrins and matrix metalloproteases, and they increase their affinity to stromal cells by expressing N-cadherin in place of E-cadherin. They thus acquire properties of mesenchymal cells that allow them to break away from the epithelial cell layer and migrate. This process is driven by five core EMT transcription factors (EMT TFs) - *SNAI1* (Snail), *SNAI2* (Slug), *TWIST1*, *ZEB1* and *ZEB2* - which are believed to be essential for its induction. EMT may be employed by cancer cells to enable local invasion and intravasation, after which they may colonise a new site, possibly by undergoing the reverse process, mesenchymal-to-epithelial transition (MET)^1–3^. In addition to metastasis, multiple studies have linked EMT to immune evasion, chemoresistance and cancer stem cell properties^4–7^.

Much has been learned about EMT in cancer using cell lines or mouse models, but despite this, its existence and potential role in human cancer progression remains unclear. This is partly because of evidence indicating that cancer cells can efficiently metastasise even without undergoing EMT^5,6,8^. However, the main reason for this uncertainty is the difficulty in detecting EMT in vivo in humans. Multiple studies suggest that EMT is transient and often partial, with cancer cells able to move dynamically and reversibly along a spectrum of intermediate states between fully epithelial and fully mesenchymal^2,3^. The term Epithelial-Mesenchymal Plasticity (EMP) was recently proposed to better describe this range of cell states^9^. Moreover, cells that have undergone EMT closely resemble normal mesenchymal cells in the tumour microenvironment (TME), especially cancer-associated fibroblasts (CAFs)^3^. CAFs constitute a major tumour component in most cancer types, and they express many mesenchymal and EMT markers.

This is particularly problematic for analysis of bulk-level expression data from human tumour samples, where findings concerning EMT could easily be confounded by the presence of CAFs. For example, large-scale expression profiling of tumours in studies of The Cancer Genome Atlas (TCGA) has uncovered mesenchymal programs in a variety of cancer types, which were the basis for the definitions of “mesenchymal” subtypes of cancers including glioblastoma, ovarian cancer and head and neck squamous cell carcinoma (HNSCC)^10–12^. However, multiple studies have demonstrated that these programs may also reflect CAFs. For example, the Mesenchymal and Basal subtypes of HNSCC differed primarily by the abundance of CAFs rather than the mesenchymal programs of the cancer cells^13^. Similarly, the mesenchymal subtype of ovarian cancer was found to be accounted for by high abundance of CAFs^14^. Other studies defined subtypes of pancreatic and colorectal cancers with mesenchymal properties^15–18^, which were later shown to also be confounded by CAFs^19–21^.

Various methods have been developed to deconvolve bulk expression profiles into constituent cell types or states^22,23^. However, these methods rely on coherent marker gene sets for well-defined cell subpopulations, rendering them inappropriate for decoupling indistinct and heavily overlapping signatures. In this study we demonstrate an approach for decoupling the mesenchymal expression profiles of cancer cells and CAFs by analysing both single cell and bulk expression profiles. We develop a deconvolution method which we apply to TCGA bulk expression data to characterise the EMT program across hundreds of tumours from 12 cancer types, while accounting for the presence of stroma. We observe expression signatures of partial EMT (pEMT) which lack strong association with the core EMT TFs, cluster into three distinct types and correlate with metastasis and other prognostic features only in specific contexts.

## Results

### Evaluating EMT and CAF expression programs by single cell RNA-seq

Single cell RNA sequencing offers an ideal setting to directly compare the expression of mesenchymal signature genes in cancer cells and fibroblasts and thereby decouple EMT and CAF programs. However, due to cost and technical challenges, most published studies of scRNA-seq in carcinomas included relatively few patient samples or sequenced few cancer cells. As a result, with the exception of HNSCC^13^, these scRNA-seq profiling studies usually did not define EMT patterns de novo and mostly either did not refer to EMT programs in cancer cells^24–30^ or used literature gene-sets as a proxy for EMT^31–33^ without assessing their coherence within the scRNA-seq data.

**Fig. 1:**
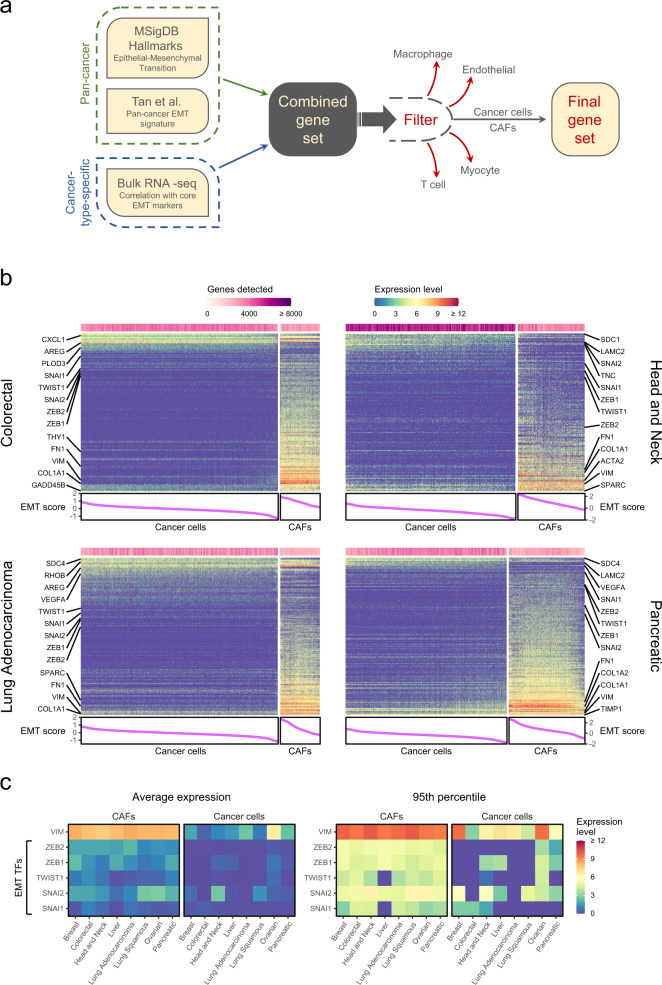
Expression of ESGs in cancer cells and CAFs by scRNA-seq. **a** Scheme depicting the gene selection and filtering process for defining cancer type-specific lists of EMT signature genes (ESGs). **b** Heatmaps showing expression levels of ESGs (rows) in cancer cells and CAFs (columns) in 4 scRNA-seq datasets (panels) out of the 8 considered (the remaining 4 are shown in Fig. S4). Columns (cells) are ordered by their EMT scores (see Methods), which are shown in the line graphs below the heatmaps. The bar above each heatmap shows the number of genes detected in each cell. **c** Heatmaps showing the average and 95^th^ percentile of the expression levels of each of the core EMT TFs and Vimentin in cancer cells and CAFs in each of the 8 scRNA-seq datasets.

To provide a comprehensive view of EMT from scRNA-seq data, we first collected data from all published studies of epithelial cancers that we deemed most relevant (Table S1). We redefined cancer cells in each of these datasets by inferring copy number alterations (CNAs), and identified non-cancer cell types (immune and stroma) by expression of marker genes, while maximising the consistency with the authors’ original classifications (see Methods) (Figs. S1–S3). Next, we defined cancer-type-specific lists of EMT signature genes (ESGs) reflecting the expected EMT signature of each cancer type. For this, we combined three gene sets which represent complementary definitions for EMT-related genes (Fig. 1a): (1) the MSigDB Hallmark EMT gene set^34,35^, which is based on consensus across many EMT-related gene sets; (2) the EMT signature genes from Tan et al.^36^, selected due to their consistency with the EMT program across many cancer types; and (3) genes that correlate highly in bulk RNA-seq data with classical EMT markers (Vimentin and the 5 EMT TFs). The latter approach was done separately for each cancer type, and then integrated with the former two pan-cancer gene-sets, thereby generating a collection of cancer-type-specific ESG lists. For each cancer type, we also excluded those genes whose expression was highest in a cell type other than cancer cells or CAFs (see Methods).

Next, we examined the expression of these ESGs in both cancer cells and CAFs in the selected scRNA-seq datasets (Fig. 1b, S4a). In all cancer types examined, the ESG signal was stronger in CAFs, which expressed more ESGs and at overall higher levels than the cancer cells. The cancer cells expressed a smaller subset of ESGs, typically at low and largely uniform levels, with some exceptions of patient-specific effects (Fig. S4b). Thus, the data provided limited evidence for EMT. Importantly, in most studies Vimentin and the EMT TFs were expressed at very low levels in the cancer cells and much more highly in CAFs, with the notable exception of *SNAI2* in HNSCC (Fig. 1c). Expression of *TWIST1* and *ZEB2* was largely absent in cancer cells, and, in 6 of the 8 cancer types, even the 95^th^ percentile of Vimentin expression levels in cancer cells was lower than its average expression level in CAFs. Furthermore, expression levels of epithelial markers largely did not correlate negatively with those of ESGs (Fig. S5). Taken together, these results suggest that most expression of ESGs in tumours reflects CAFs and that the weaker signal for a subset of ESGs in cancer cells is more consistent with only a partial EMT (pEMT) rather than full EMT. Notably, the expressed ESGs appear to differ between cancer types, underscoring the context specificity of potential pEMT. These results were robust to ambiguity in the definition of CAFs (Fig. S3, S6) and to the normalisation method used to define expression levels (Fig. S7).

However, given the limited numbers of profiled cancer cells and potential biases in scRNA-seq profiling, as particular cell types may not survive through single cell isolation protocols, we cannot exclude the possibility that rare cells with full EMT have escaped detection in those datasets. Moreover, the low number of tumour samples with available single cell data precludes statistical associations of the identified pEMT programs with clinical features. In contrast, bulk RNA-seq data does not suffer from the same biases associated with isolation of particular cell types, and is much more plentiful, with large and clinically annotated bulk RNA-seq datasets together comprising thousands of tumour samples. This motivates the development of a method to separate the cancer-cell-specific pEMT program from CAF signatures in bulk expression profiles.

### Simulation of bulk expression profiles indicates that mesenchymal signatures are dominated by CAFs

**Fig. 2:**
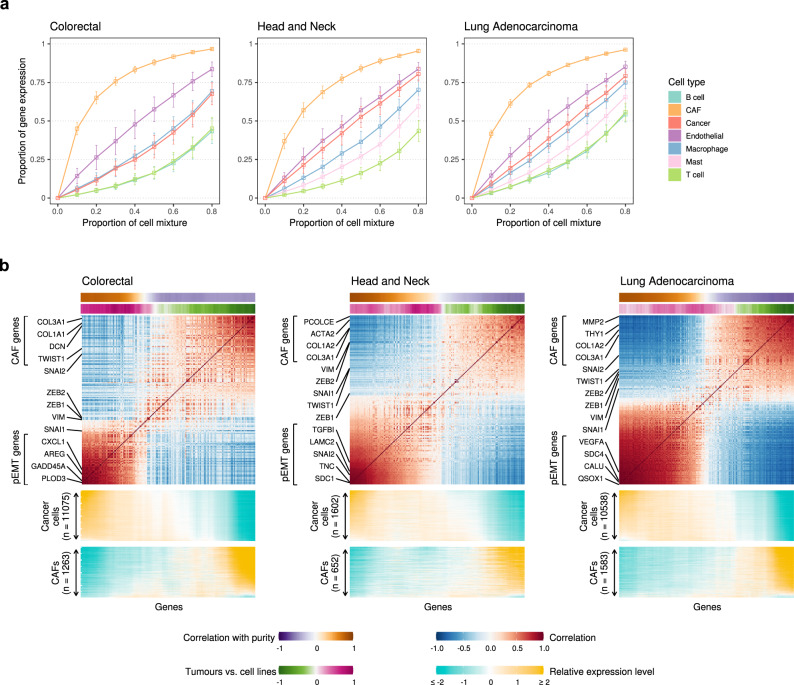
Contribution of different cell types to expression of ESGs in simulated bulk tumours. **a** Line plots showing the relative contributions of different cell types to ESG expression (EMT signal), for various fractions of tumour composition, in simulated bulk expression profiles based on 3 scRNA-seq datasets out of the 8 considered (the remaining 5 are shown in Fig. S8a). Each point represents the average proportion of EMT signature gene expression coming from the corresponding cell type in a collection of 100 simulated tumours with the given fraction of that cell type and varying proportions of the other cell types. Error bars show the standard deviation over the set of 100 simulations. **b** ESG co-expression matrices derived from simulated bulk expression profiles based on the 3 scRNA-seq datasets shown in (**a**) (the remaining 5 are shown in Fig. S8b), ordered by the SPIN side-to-side algorithm^37^ with slight modifications (see Methods). ESGs are annotated with two colour-coded panels at the top: (1) correlations with simulated tumour purity (Pearson correlation coefficient); and (2) comparison of expression levels in simulated tumours versus in cell lines, where positive numbers indicate higher expression in tumours than in cell lines. Heatmaps below the co-expression matrices show the relative expression levels of ESGs in individual CAFs (bottom rows) and cancer cells (top rows) in the scRNA-seq data. Selected ESGs are labelled at the side of each co-expression matrix. Source data are provided as a Source Data file.

Before analysing bulk expression profiles, we sought to understand the expected expression patterns of ESGs in bulk profiles based on their patterns in scRNA-seq data. We simulated bulk expression profiles by sampling from the scRNA-seq data various fractions of CAFs and cancer cells, as well as other annotated cell types, and aggregating their individual expression profiles. We thus obtained a set of bulk expression profiles for simulated tumours with known cell type composition and corresponding single-cell expression data, which enabled precise calculation of the contribution of each cell type to the bulk expression of ESGs. We evaluated this contribution for each cell type by averaging over all simulations in which that cell type had a particular cellular frequency (Fig. 2a, S8a).

The contribution of CAFs to the bulk ESG signal was consistently high in all the cancer types considered and in a range of simulated tumour compositions. For example, in every cancer type, when simulated tumours contained 30% CAFs, these CAFs accounted, on average, for well over half the total ESG signal, and hence more than the cancer cells. Notably, other components of the tumour microenvironment, especially endothelial cells and macrophages, also contributed significantly to the total ESG signal, such that in many tumour compositions their contributions were comparable to or higher than that of the cancer cells.

### Partial EMT profiles may be decoupled from CAF profiles in simulated bulk expression profiles

While the mesenchymal signal in simulated bulk expression profiles predominantly reflects the presence of CAFs, our analysis also identifies specific ESGs (e.g. *SNAI2*, *SDC1/4* and *LAMC2*) whose expression levels in the cancer cells are higher than or comparable to those in CAFs (Fig. 1b). These might still be associated with metastasis, either by directly promoting cell migration or by serving as an intermediate step towards a rare or transient full EMT that promotes metastasis but was not captured in the scRNA-seq data. Although expression of ESGs in bulk samples reflects the sum of their expression across multiple cell types, we reasoned that ESGs expressed highly by cancer cells would have a different co-expression pattern from those expressed primarily by CAFs, and hence that co-expression patterns would enable deconvolution of the bulk ESG signal. The assumptions behind this approach are that: (1) CAF profiles and pEMT profiles are similar between patients of a given cancer type (or subtype); but (2) the fractions of CAFs and of pEMT cells vary significantly between tumours, and the correlation between these two fractions is limited. Hence, the correlations among cancer-cell-enriched ESGs, and likewise among CAF-enriched ESGs, should be significantly higher than the correlations between these two groups of genes. We thus expect to be able to separate ESGs into two groups representing pEMT and CAF signatures using bulk expression profiles.

To test this with the simulated bulk expression data, for each cancer type we constructed a co-expression matrix for a subset of ESGs, excluding those genes which correlated highly with marker genes for non-malignant cell types other than CAFs (see Methods). Ordering ESGs by their co-expression pattern using the SPIN algorithm^37^ (with slight modifications) revealed a separation into two clusters that closely mirrors the relative expression of ESGs by cancer cells (left cluster) and by CAFs (right cluster) (Fig. 2b, S8b). Additional ESGs (around the midpoint of the co-expression matrix) are not clearly associated with either one of the clusters as they co-vary with both pEMT and CAFs to a similar degree. Interpretation of these clusters was validated by examining the relative expression levels of ESGs in individual cancer cells and CAFs in the scRNA-seq data (Fig. 2b, S8b, lower panels).

We examined two additional measures of association with cancer cells to enable the decoupling approach when scRNA-seq data is not available (Fig. 2b, S8b, top panels). First, we calculated the correlation of each gene with tumour purity (the fraction of malignant cells), which is known for our simulated tumour cohort but in general may be estimated from bulk tumour DNA profiles^38^. As expected, purity correlations were higher for the cancer cell cluster of genes than for the CAF cluster. Second, we defined relative expression of genes in a collection of cancer cell lines^39^ compared to tumours (see Methods). As cell lines contain only cancer cells, while tumours comprise a heterogeneous microenvironment, higher expression in cell lines suggests an enrichment with cancer cells. This measure was also much higher for the cancer cell cluster of genes than for the CAF cluster, with minor discrepancies observed in liver and lung squamous cell carcinomas. Such discrepancies may arise because cell lines only partially recapitulate the in vivo state of cancer cells and may express cellular programs not seen in human disease. Thus, this measure is only used as a secondary validation and not as an integral element of the method. Overall, these three distinct validation approaches, incorporating scRNA-seq, tumour purity and cancer cell lines data, converge to a consistent annotation of ESG clusters as reflecting pEMT and CAF expression programs.

### Co-expression patterns in bulk TCGA profiles recapitulate pEMT and CAF separation

**Fig. 3:**
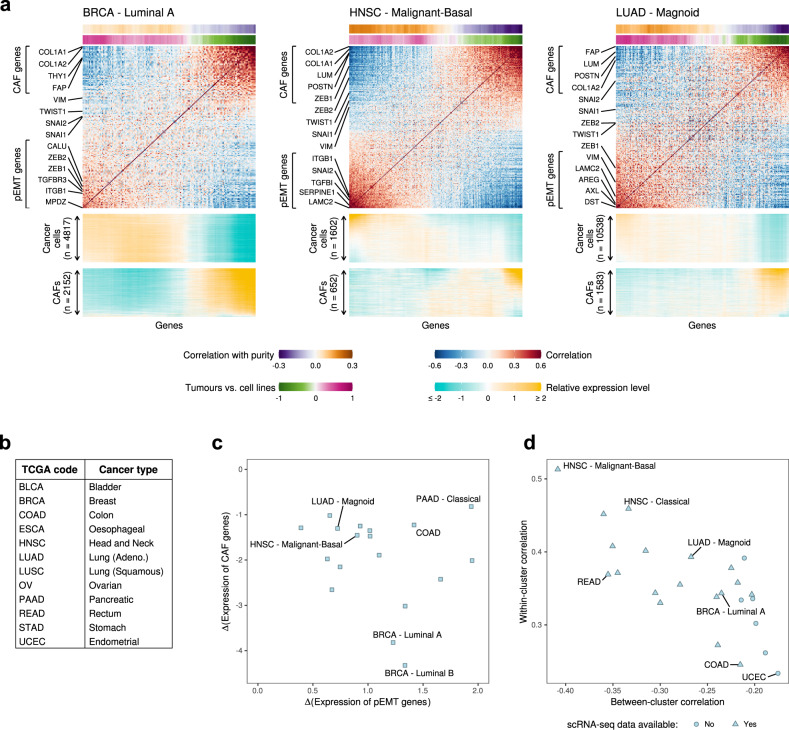
Deconvolution of cancer and CAF ESG expression from TCGA bulk expression profiles. **a** ESG co-expression matrices derived from TCGA bulk expression data for 3 cancer types of the 24 considered (the remainder are shown in Fig. S10), ordered by the SPIN STS algorithm^37^ with slight modifications (see Methods). ESGs are annotated with two colour-coded panels at the top: (1) Pearson correlations with estimates of tumour purity computed by ABSOLUTE^38^; and (2) comparison of expression levels in tumours versus in cell lines, where positive numbers indicate higher expression in tumours than in cell lines. Heatmaps below the co-expression matrices show the relative expression levels of ESGs in individual CAFs (bottom rows) and cancer cells (top rows) in the relevant scRNA-seq dataset. Selected ESGs are labelled at the side of each co-expression matrix. Source data are provided as a Source Data file. **b** Table of cancer types corresponding to the TCGA disease codes. **c** Summary scatterplot of the deconvolution results for the 18 cancer types having accompanying scRNA-seq data, showing the difference in average relative expression levels of the top 20 pEMT genes (X-axis) and the top 20 CAF genes (Y-axis) between cancer cells and CAFs. Positive values indicate higher expression in cancer cells and negative values indicate higher expression in CAFs. **d** Summary scatterplot of the deconvolution results for all 24 cancer types examined, showing for each cancer type the average correlations among genes from the same cluster of ESGs (within-cluster, Y-axis) and between genes from the two different clusters of ESGs (between-cluster, X-axis).

We next applied the above deconvolution method to TCGA bulk RNA-seq datasets for multiple epithelial cancer types with available scRNA-seq profiles. For those cancer types with a large number of samples and well-established subtype definitions, we examined each subtype separately in order to consider the possibility of subtype-specific pEMT profiles and to minimise the influence of differences between subtypes on the deconvolution results. In a total of 22 cancer subtypes, we obtained a separation of ESGs into two clusters that appear to reflect cancer cells and CAFs. To confirm this interpretation, we examined the three annotation measures described above - relative expression in cancer cells vs. CAFs by scRNA-seq, correlation with tumour purity and expression in tumours vs. cell lines. Two cancer types were excluded from further analysis due to insufficient support from these measures, and a further two were excluded due to potential confounding effects by non-malignant cells besides CAFs (Fig. S9). For each of the remaining 18 cancer types/subtypes, we obtained a robust separation of ESGs into two clusters that were annotated as pEMT and CAF programs based on the three validation measures (Fig. 3a,b, S10a). It is important to note that the scRNA-seq data used for annotation of ESG clusters was not used in the deconvolution method itself, hence this measure constitutes an independent validation of the inferred pEMT and CAF signatures (Fig. 3c).

Given the consistency between these three measures for assigning ESG clusters to CAFs and cancer cells (Fig. S9a), we reasoned that we could also provide putative annotations for ESG clusters based only on the latter two methods, thereby relieving the requirement for scRNA-seq data. We thus extended the analysis to epithelial cancer types without scRNA-seq datasets. This allowed us to define a robust separation of ESGs into clusters which were annotated as putative pEMT and CAF signatures for 6 additional cancer types/subtypes, after filtering as described above (Fig. S9, S10b). Overall, we defined a deconvolution of ESGs in 24 cancer types/subtypes (18 with and 6 without scRNA-seq data) (Fig. 3d, S10). We note, however, that the first measure of scRNA-seq is the most reliable one (as it directly validates expression in cancer cells and CAFs) and hence the most reliable deconvolution signatures are those from cancer types/subtypes with scRNA-seq data.

### Shared and unique aspects of pEMT programs across cancer types

**Fig. 4:**
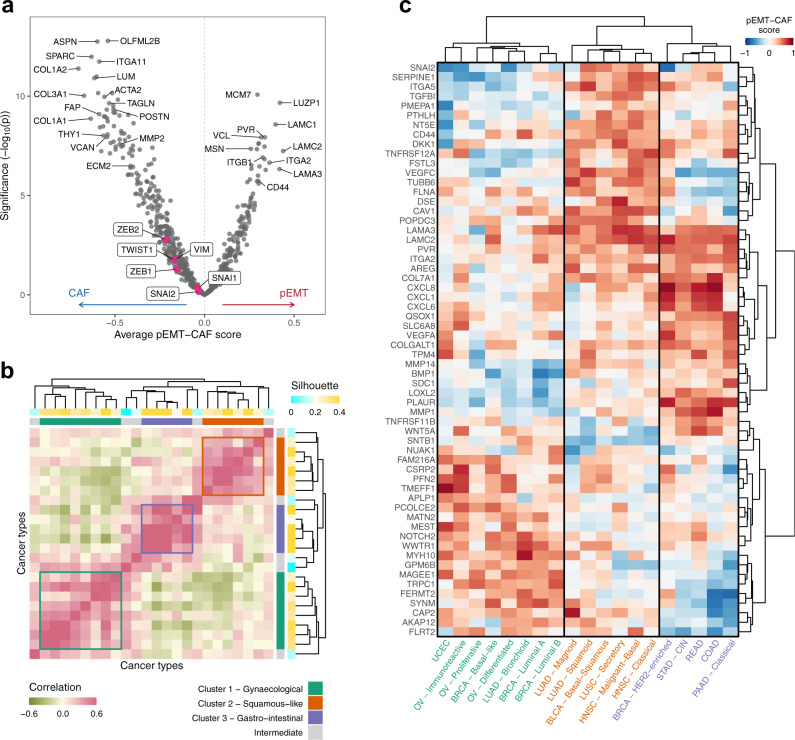
Variability of ESG association with cancer cells and CAFs across cancer types/subtypes. **a** Volcano plot showing each gene’s average pEMT-CAF score (X-axis) and its significance (Y-axis, quantified as -log10(p-value) based on two-sided T-test, without adjustment for multiple comparisons). Shown are all ESGs included in the TCGA deconvolution analysis, and selected ESGs are labelled. Source data are provided as a Source Data file. **b** Heatmap showing hierarchical clustering of pairwise correlations between cancer types/subtypes based on their pEMT-CAF scores for the 100 genes most commonly appearing in the inferred pEMT signatures, annotated by two coloured bars on each axis: (1) the silhouette of each cancer type with respect to the three largest clusters; and (2) the final cluster assignments after identifying intermediates. **c** Heatmap of pEMT-CAF scores for the top 20 differentially expressed pEMT genes in each of the three cancer type clusters. Both axes are ordered by hierarchical clustering. Source data are provided as a Source Data file.

Next, we assessed the consistency of pEMT and CAF programs across the 24 cancer types/subtypes. We defined a score for each gene in each cancer type/subtype, reflecting its relative co-expression with the pEMT ESGs compared with the CAF ESGs (Fig. S11). Averaging these scores across cancer types/subtypes (Fig. 4a) identified many ESGs that are strongly and consistently associated with the CAF signatures, including classical CAF markers such as *FAP*, *SPARC*, *THY1* (*CD90*) and various collagens. In contrast, relatively few genes were consistently associated with the pEMT program across cancer types, and these were not as strongly biased as the CAF genes. This suggests that pEMT programs exhibit greater context-specificity than the CAF signatures, underscoring the difficulty in defining universal pEMT markers. Notably, Vimentin and the five EMT TFs were all weakly biased towards CAFs, with none showing a consistent association with pEMT. Instead, the top pEMT-associated genes included laminins (*LAMC1*, *LAMC2*, *LAMA3*), integrins (*ITGA2* and *ITGB1*), *CD44* and *PVR*. However, even these genes are differentially associated with pEMT across cancer types/subtypes (Fig. S11).

To further characterise the context-specificity of pEMT programs, we computed the pairwise correlations between cancer types with respect to their ESG scores (Fig. 4b). This analysis identified three clusters of cancer types, as well as some cancer types that appeared as intermediates. This variability is not fully explained by global differences between cancer types/subtypes but instead is partially specific to the pEMT profiles  (Fig. S12). Interestingly, these three clusters were primarily, though not exclusively, associated with gynaecological (breast, ovarian and endometrial), squamous cell or “squamous-like” (HNSCC, lung and bladder “basal-squamous”) and gastro-intestinal (colorectal, stomach and pancreatic) carcinomas, respectively. Many of the ESGs were differentially associated with pEMT between the three clusters (Fig. 4c). For example, *NOTCH2*, *WWTR1* (*TAZ*) and *MYH10* were preferentially associated with pEMT in the gynaecological cluster, while *TGFBI*, *ITGA5* and *SNAI2* were preferentially associated with pEMT in the squamous cluster. This separation into three clusters suggests the existence of distinct versions of pEMT that may reflect the cancer’s cell of origin and/or the influence of its microenvironment.

### Association of pEMT and CAFs with clinical features

**Fig. 5:**
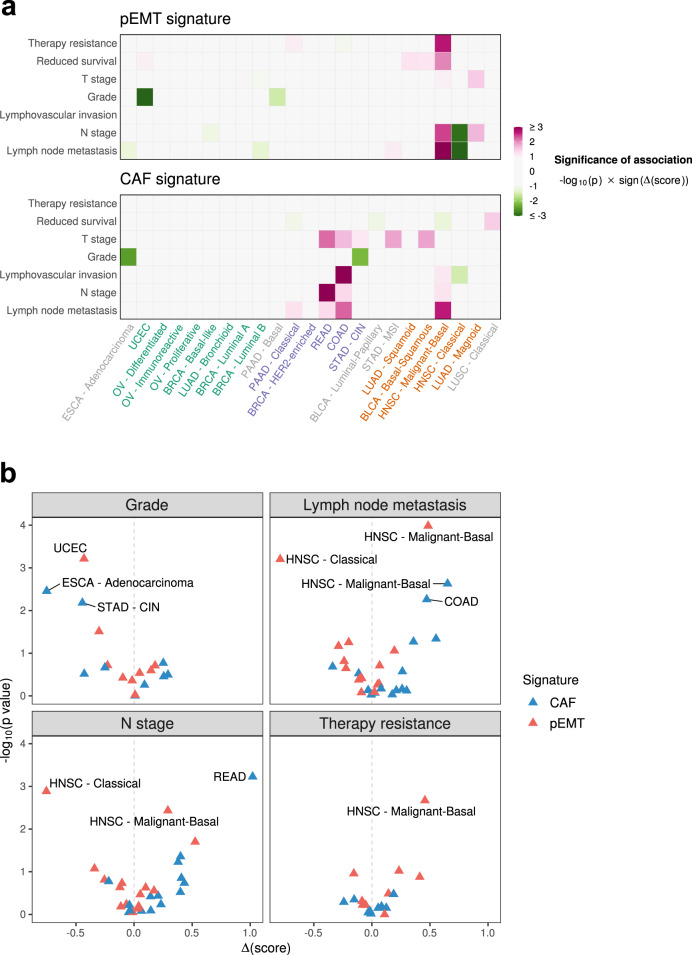
Association of pEMT and CAF signatures with clinical features. **a** Heatmaps showing the significance (quantified as –log10(p-value) based on a two-sided Wilcoxon rank-sum test, without adjustment for multiple comparisons) of the association of signatures for pEMT (top) and CAFs (bottom) with seven clinical features (rows) reflecting worse prognosis, in 23 of the 24 cancer types (columns) passing quality control (the LUSC Secretory subtype is absent due to low sample size). Positive and negative associations are depicted in purple and green, respectively. The cancer types are ordered by hierarchical clustering of their pEMT-CAF scores, and coloured by their pEMT cluster assignments. **b** Volcano plots showing, for each of four clinical features, the significance (Y-axis, defined as in **a**) of its association with pEMT signatures (red) and with CAF signatures (blue) against the effect size (X-axis, quantified as the difference in signature score). Points are labelled with their corresponding cancer types if they pass an adjusted significance threshold corresponding to an FDR of 0.05. Source data are provided as a Source Data file.

Next, we evaluated the statistical association between average expression of pEMT and CAF signature genes and seven clinical features available for TCGA samples, including lymph-node metastasis, tumour grade, survival time and therapy resistance (Fig. 5, S13a). Distant metastasis could not be included in this analysis due to low sample number (see Methods), as primary tumours are typically not surgically removed when distant metastases are observed. In general, most clinical associations were not significant, and there were no coherent associations across cancer types, suggesting that the clinical significance of pEMT (and CAF) expression programs may be complex and highly context-specific.

However, a few significant associations stood out. First, consistent with previous work^13^, lymph-node metastasis, as well as the related N-stage, had a highly significant association with pEMT in the malignant-basal subtype of HNSCC. Surprisingly, similar associations were not observed for other cancer types and subtypes, suggesting that the extent of pEMT may be particularly important for metastasis in HNSCC. We also observed an unexpected negative association of pEMT with lymph-node metastasis and N-stage in the classical subtype of HNSCC. However, this association may be confounded by a broader separation of the classical subtype into two groups, which differ by pEMT as well as other expression programs (Fig. S13b).

Second, pEMT was also strongly associated with therapy resistance in malignant-basal HNSCC. Third, the CAF signatures also exhibited clinical associations. CAF associations were almost entirely distinct from those of pEMT, but primarily involved three clinical features associated with invasion or metastasis (lymph-node metastasis, N-stage and lymphovascular invasion). Such associations could previously have been misinterpreted as reflecting pEMT, highlighting the importance of decoupling these two sources of ESG expression.

## Discussion

In this study, we developed an integrative approach to decouple the stromal mesenchymal signature from true cancer-cell EMT programs across many cancer types. The inferred EMT signatures would benefit from orthogonal validation, such as in situ staining of tumour sections. However, such methods have specific limitations and are typically focused on individual genes rather than on entire gene-set signatures.

Our analyses reveal several important properties of EMT that should be accounted for in future research. Firstly, the EMT signatures we derived from both single-cell and bulk expression data are partial, with expression of classical EMT markers either missing or more dominant in CAFs. In particular, with the exception of *SNAI2*, the EMT TFs are typically expressed at comparable or higher levels by CAFs and should not be used as markers for pEMT in bulk expression profiles. Secondly, the pEMT program is highly context-specific, in particular differing between squamous-like, gastro-intestinal and gynaecological cancer types. This serves as a warning against using any single EMT signature for all cancer types. Additional work could help to identify the potential physiological origin of these distinct pEMT programs.

Thirdly, in most cancer subtypes, pEMT was not significantly associated with any of the examined clinical features. Among the few cases where the pEMT signature was associated with clinical features, these features varied between lymph-node metastasis, grade and therapeutic resistance. It is particularly striking that association of pEMT with lymph-node metastasis is largely unique to malignant-basal HNSCC. These findings suggest that pEMT may not be the main bottleneck for metastasis, which depends on many other properties. For example, the ability of cells to adapt to new niches and seed new tumours, rather than to attain the mesenchymal state required to reach the niche, may be the true bottleneck for metastasis^40^. Further complicating this picture is the idea that collective migration may be more effective at seeding metastases than dissemination of lone cells^41^. It is also possible that lymph-node metastasis is a poor approximation for distant metastasis, and that the pEMT program may still be a strong predictor for distant metastasis. Additionally, dissemination of cancer cells may occur very early in tumour development^42^, even before diagnosis of the primary tumour, such that the observation of pEMT in primary tumour samples would be independent of the presence of metastases.

In this work we focused on decoupling ESG expression into two main components that largely correspond to CAFs and cancer cells. However, our analysis also points to significant contributions of other cell types to mesenchymal expression profiles, most notably endothelial cells and macrophages. Moreover, for any individual cell type there is considerable cellular heterogeneity that is largely ignored by our approach. This is certainly true for CAFs, which may exist in resting, myofibroblastic, inflammatory and antigen-presenting forms, and may promote or inhibit tumour development^43,44^. These complications are expected to have a limited influence on the global inference from co-expression patterns across hundreds of bulk tumours, but they will have a larger influence when attempting to infer the origin of ESG expression in individual patients. Thus, while deconvolution of bulk samples provides a good starting point to define pEMT patterns and their functional significance, single cell approaches would ultimately be needed to provide a more accurate and complete characterisation of individual tumour ecosystems.

Even when ESG expression is known to reflect cancer cells (rather than CAFs or other stromal cells) it is important to distinguish between two potential sources. A bulk EMT-like expression profile may reflect a small subpopulation of cells that undergo pEMT and differ from the majority of cancer cells in the same tumour (that is, rare ESG expression). Yet, it may also reflect a “baseline” expression, in which all or most cancer cells in the tumour express ESGs (common ESG expression). Rare and common ESG expression would in turn lead to intra-tumour and inter-tumour heterogeneity in ESG expression, respectively, and both of these could contribute to the patterns we observe in bulk profiles. ScRNA-seq data provides evidence for both rare and common ESG expression. For example, many of the cancer cells in HNSCC express *SNAI2*, while only few of those cells express a full pEMT program that includes *TGFBI*, *SERPINE1* and other ESGs, but not higher *SNAI2* levels^13^. Our observation of *SNAI2*, *TGFBI* and *SERPINE1* in the pEMT signatures of squamous-like cancer types (Fig. 4c) suggests that these signatures reflect both inter-tumour and intra-tumour heterogeneity. It is not clear which of these is most relevant to cancer development and metastasis and future studies would be needed to further evaluate this.

In summary, this study presents a pan-cancer characterisation of pEMT signatures and their association with clinical features, decoupled from stromal confounders. Our findings demonstrate the predominantly partial nature of EMT, as well as its context-specificity and its limited but varied connection with prognostic features. These results help to explain the controversy and ambiguity concerning EMT, while highlighting multiple avenues for further research and providing guidance on where future EMT work should focus.

## Methods

All computational analysis for this study was performed using R version 3.6.3 (“Holding the Windsock”). The individual R package version numbers can be found in the source code, which is available at https://github.com/m20ty/decoupling_emt_stroma.

### Preparation of scRNA-seq data

The 8 scRNA-seq datasets used in this study are described in Table S1. These datasets were taken in their publicly available processed forms. Gene names were mapped to the most up-to-date HGNC symbols using either the limma or AnnotationDbi packages in R. Genes which could not be unambiguously mapped in this way were removed. Following this, cells with fewer than 1000 genes detected were removed. In the HNSCC dataset, only 10 of the 18 tumour samples were used in the analysis, namely those identified in the original publication as having the most malignant cells^13^. For the main analysis, expression levels were converted to log_2_(TPM_*i,j*_/10 + 1), where TPM_*i,j*_ refers to the transcripts-per-million estimate for gene *i* in sample *j*. The TPM values were divided by 10 in every dataset to reflect an upper bound estimate of 100,000 for the number of transcripts in single-cell libraries. The same estimate was used for all datasets to enable comparability between them. A parallel analysis was also performed on three datasets normalised using the scran R package^45^, which demonstrated that the analysis is robust to the normalisation method.

### Inference of copy number alterations (CNAs)

To infer CNAs from the scRNA-seq data, we first defined putative non-malignant cells by using t-SNE to reduce the data to two dimensions, then applying DBSCAN to define clusters of cells in the t-SNE space. The clusters were assigned to cell types by examining the expression of marker genes. CNAs were then computed via an approach described previously^46,47^, using the infercna R package (https://github.com/tiroshlab/infercna). Briefly, genes were ordered by their chromosomal location and a moving average of their relative expression levels was computed, using a sliding window of 100 genes. The resulting CNA values were then adjusted relative to those of the putative non-malignant cell types identified as above. Note that only immune and endothelial cells were used as reference cells to adjust the CNA values in this way. Putative CAFs were excluded from the reference in order to avoid bias in later analysis distinguishing cancer cells from CAFs.

### Identification of cell types

Malignant cells were identified using CNA values in a two-step process. Firstly, in each sample we applied hierarchical clustering to the CNAs of all non-reference cells, and clusters having visibly high CNAs were manually annotated as putative malignant cells. In samples with multiple distinct clusters of high-CNA cells, these clusters were annotated as putative subclones. Samples with no clear high-CNA cluster, or for which the high-CNA clusters together contained fewer than 50 cells, were excluded from further analysis. Secondly, for each cell we defined the “CNA signal” as the mean of the squares of its CNA values, and the “CNA correlation” as the Pearson correlation of its CNA profile with the average CNA profile of the putative malignant cells identified in the first step. Where relevant, the CNA correlation was calculated separately for each subclone. For each tumour/subclone, we manually chose thresholds separating cells with low CNA signal and correlation from those with distinctly higher values for both measures. The former were designated as non-malignant and the latter as malignant, and any intermediates were excluded from further analysis. We further excluded cells for which our malignant/non-malignant classification disagreed with the original publication from which the dataset was obtained, in order to arrive at a consensus.

We next applied t-SNE and DBSCAN to the non-malignant cells to identify clusters, which were assigned to cell types by examining expression of marker genes. Where these cell type classifications broadly agreed with those from the original publication, the latter were used in downstream analysis. Where there was substantial disagreement, our own classifications were used. Cells which we were unable to identify were excluded. Particular attention was paid to the CAFs: in cases where multiple distinct clusters resembled CAFs, we used t-tests to identify genes differentially expressed between them. If one such cluster preferentially expressed genes not typically associated with CAFs, or genes associated with pericytes, these cells were labelled as “potential CAFs”. These potential CAFs were excluded from the main analysis, but were included as part of the CAF definition in a parallel analysis that demonstrated the robustness of the approach to the precise definition of CAFs. A final t-SNE was applied to all malignant and non-malignant cells together, and any malignant cells appearing in a non-malignant cell cluster, or vice versa, were excluded from further analysis.

### Preparation of TCGA bulk expression data

The TCGA bulk RNA-seq datasets for the cancer types considered in this study were downloaded from the Broad GDAC Firehose website (http://gdac.broadinstitute.org/). The cancer types considered included all epithelial cancers having at least 100 samples. The expression levels were defined as log_2_((*E*_*i,j*_  × 10^6^) + 1), where *E*_*i,j*_ refers to the RSEM^48^ scaled estimate for gene *i* in sample *j*. Briefly, the RSEM algorithm estimates transcript abundances from RNA-seq reads using a directed graph model and the expectation maximisation algorithm. The resulting estimates are returned both as transcript “counts” (which are typically non-integer) and as “scaled estimates”, which are scaled to the total number of transcripts in the sample and which, if multiplied by 10^6^, measure the abundances in terms of TPM. Gene IDs were mapped to the most up-to-date HGNC symbols using the AnnotationDbi package in R. Genes whose IDs could not be mapped to symbols in this way were removed. Samples derived from normal (non-tumour) tissue were excluded.

### Selection of EMT signature genes (ESGs)

The ESGs were obtained by combining gene sets from three sources: the MSigDB Hallmarks “Epithelial-Mesenchymal Transition” gene list^34,35^; the EMT signature genes from Tan et al.^36^ (the union of the signatures for tumours and those for cell lines); and genes that correlate highly with the 6 classical EMT markers *SNAI1*, *SNAI2*, *TWIST1*, *VIM*, *ZEB1* and *ZEB2* in bulk expression data from TCGA. The third approach is specific to each cancer type and was undertaken as follows. For each cancer type and its corresponding TCGA bulk RNA-seq dataset, we calculated the average correlation of each gene in this dataset with the above 6 classical EMT markers. From the distribution of these averages, the genes comprising the highest 1% were defined as ESGs. For each cancer type, the former two gene lists were combined with the third, cancer-type-specific list to define the set of all ESGs for that cancer type. To define ESGs to be used in the analysis of an scRNA-seq dataset, the TCGA bulk RNA-seq data for the corresponding cancer types was used (Table S1).

### Definition of gene signature scores in scRNA-seq data

Scores for a given gene signature were computed for individual cells to quantify the degree to which they express the signature genes. These scores were defined using an approach described previously^47^, in which the expression of each signature gene is measured relative to a control gene set. These control gene sets are chosen to recapitulate the distribution of expression levels among the genes in the signature, while having no coherent association with any particular cellular program. This method is designed to alleviate the possible influences of complexity (that is, number of genes detected per cell) on association of cells with a gene signature, since cells with higher complexity would be expected to have high average expression of any set of genes. It also lessens the impact of genes with especially high expression or variance, which may otherwise dominate the signature score.

The method proceeds as follows. For a given scRNA-seq dataset and a given subset of cells (e.g. the cells in a chosen tumour), we take the subset of genes passing some criteria on minimal expression level (depending on the particular analysis and dataset). These genes are ordered by their average expression levels across cells and partitioned into *m* bins (each of which may contain some genes from the signature of interest). For each gene *G*_*j*_ in the signature, a set of *n* genes {*G*^0^_*k*_}_*k*=1,…,*n*_ is randomly sampled from the same expression bin as *G*_*j*_ (this sample will contain mostly random genes, but may by chance also include some signature genes). We then compute the relative expression level *G*_*j*_^rel^(*i*) of *G*_*j*_ in cell *i* as *G*_*j*_(*i*) – mean({*G*^0^_*k*_(*i*)}_*k*=1,…,*n*_), and the score SC(*i*) for cell *i* as mean({*G*_*j*_^rel^(*i*)}_*j*_). The parameters *m* and *n* vary depending on the particular analysis and dataset.

### Analysis of expression of mesenchymal signature genes in scRNA-seq data

For each scRNA-seq dataset, we selected a set of ESGs as described above and applied additional filtering as follows. Firstly, for each annotated cell type, we calculated the average expression of each ESG across cells of that type. We then removed those ESGs whose average expression was highest in a cell type other than cancer cells or fibroblasts, resulting in between 230 and 360 ESGs. Secondly, we filtered the remaining genes by expression level, retaining only those genes with average TPM value above a chosen threshold (depending on the specific dataset), the average being taken across cancer cells and fibroblasts. This filtered ESG list, comprising between 120 and 250 genes, is exactly the set of genes whose expression levels are depicted in the heatmap for the corresponding cancer type in Fig. 1b, S4. From this filtered ESG list, we chose an EMT signature to be used for cell scoring by taking those genes with average TPM value above a stricter threshold (depending on the dataset), the average being taken across only the cancer cells. We scored the cancer cells and fibroblasts for this EMT signature as above, calculating scores separately within each tumour to mitigate the effects of inter-tumour heterogeneity. We then ordered the cells in the heatmap by the resulting EMT score. This ordering highlights the more mesenchymal cells, while controlling for the effect of complexity.

### Comparison of epithelial marker gene expression with EMT score

We defined an initial set of epithelial marker genes consisting of *CDH1*, *EPCAM*, *SFN,* and all keratins, then, in each scRNA-seq dataset, we retained only those from the initial list having either: (1) average TPM value above a chosen threshold (depending on the dataset); or (2) very high expression (defined as log_2_(TPM/10 + 1) ≥ 7) in at least 1% of cells. These filtered epithelial marker lists were also used as signatures to compute epithelial scores, as described above. These gene sets and the corresponding scores are displayed in Fig. S5. The expression levels shown in these heatmaps are relative expression levels, defined, for gene *i* and cell *j*, as *Z*_*i*,*j*_ = [*E*_*i*,*j*_ – mean({*E*_*i*,*j*_}_*j*_)]/std({*E*_*i*,*j*_}_*j*_), where std denotes the standard deviation. EMT scores were calculated as before, with minor modifications. The correlations of epithelial marker genes with these EMT scores were calculated separately within each tumour and then averaged across tumours, in order to mitigate the effects of differences between tumours.

### Simulation of bulk expression profiles

Bulk expression profiles were simulated by aggregating expression profiles for single cells in scRNA-seq datasets. The cells to be aggregated were sampled randomly from the dataset, with counts for each cell type depending on the desired cellular composition of the simulated tumour. To examine the relative contributions of the various cell types to the ESG signal, we simulated bulk profiles on a per-cell-type basis – briefly, the counts for one cell type were drawn from a normal distribution with suitably chosen mean and standard deviation, and those for the other cell types were chosen from a uniform distribution and scaled to give the desired proportion of the initial cell type. This approach was utilised in spite of the proportions of different cell types occurring in the scRNA-seq datasets and the cellular compositions of the samples, as biases associated with capture of particular cell types during the scRNA-seq protocol preclude an accurate estimation of the true cell type proportions.

For each dataset, we chose a threshold for minimum cell type count, and for cell types having counts below this threshold, we changed their annotations to a combined “rare cell types” category to avoid their overrepresentation in simulated tumours. (Note the cell types in this “rare” category differ between datasets.) For each cell type *C*_*i*_ (except the “rare cell types” category), and for each proportion *q*_*j*_ in the set *Q* = {0.1, 0.2, …, 0.8}, we simulated 100 tumours having approximately proportion *q*_*j*_ of this cell type. We did this by first sampling 100 counts from a normal distribution with mean *µ*_*j*_ = *µq*_*j*_/max(*Q*) and standard deviation *σ*_*j*_ = 0.2*µ*_*j*_, where *µ* is manually chosen to represent a reasonable maximum average cell type count (based on the cell type counts in the data). For each such count *N*_*i*_, we then chose counts {*N*_*k*_}_*k* ≠ *i*_ for the remaining cell types {*C*_*k*_}_*k* ≠ *i*_ (including the “rare cell types” category) by sampling values {*N*^unif^_*k*_}_*k* ≠ *i*_ from a uniform distribution with minimum 0 and maximum *µ*, scaling these values by a factor *λ* so that *N*_*i*_/(*N*_*i*_ + *λ* ∙ ∑_*k* ≠ *i*_ *N*^*unif*^_*k*_) = *q*_*j*_, then rounding the resulting values to their nearest integers: *N*_*k*_ = round(*λN*^*unif*^_*k*_). For each of the resulting counts, cells of the corresponding cell type were sampled (with replacement) at random from the dataset.

The selected ESGs for each cancer type were filtered as before based on having highest average expression in either cancer cells or fibroblasts, resulting in between 230 and 360 genes per cancer type. The contribution of the initial cell type *C*_*i*_ to the overall ESG signal was then defined as the fraction of the total expression of ESGs in the cells sampled for cell type *C*_*i*_ out of the total ESG expression in all sampled cells.

A similar approach was used to simulate bulk expression profiles for deconvolution, but here, only the cancer cells were used as an “initial” cell type to be sampled from a normal distribution. For each proportion *q* in the set *Q* consisting of the 40 values spaced equally in the range 0.1 to 0.9 (inclusive), we sampled 25 counts using a normal distribution as before, assigning these counts to the cancer cells. For each such count, we then chose counts for all other cell types (including the “rare cell types” category) by sampling values from a uniform distribution and scaling as above so that the proportion of cancer cells in the resulting tumour would be equal to *q*. For each of the resulting counts, cells of the corresponding cell type were sampled at random (with replacement) from the dataset. The simulated bulk tumour profile was then defined as the vector of elements log_2_(∑_*m*_ TPM_*l*,*m*_/10 + 1), where TPM_*l*,*m*_ refers to the transcripts-per-million estimate for gene *l* in cell *m*.

### Deconvolution of cancer and CAF ESG expression from bulk expression profiles

Estimates of tumour purity by ABSOLUTE were obtained from several sources^38,49,50^, and duplicate estimates for any given sample were averaged. For each cancer type, we took the subset of the TCGA bulk RNA-seq data consisting of those samples for which we had obtained purity estimates. If CCLE or scRNA-seq data were available for this cancer type, we took the intersection of the corresponding cancer-type-specific ESG list (chosen as above) with the sets of genes appearing in these additional datasets. Tumour subtype assignments were obtained from various sources^12,50–56^.

Since we wished to focus on the ESG signal from cancer cells and CAFs, in some cancer types we further filtered the ESG list to alleviate the possible confounding effects of other cell types of the TME on the deconvolution result. The cell types we considered included B cells, B plasma cells, dendritic cells, endothelial cells, macrophages, mast cells, myocytes and T cells. For each cell type *C*_*i*_ from this list, we manually curated a set of marker genes for *C*_*i*_ and used these to compute a weight *W*_*i*_ reflecting the overall correlation of *C*_*i*_ with ESGs and thus its likelihood of confounding the separation of ESGs into cancer and CAF components. Specifically, for each ESG *g* and each cell type *C*_*i*_, we computed the average correlation *ρ*_*g*,*i*_ of *g* with marker genes for *C*_*i*_. We then defined *W*_*i*_ as a quantile *q* of the distribution of these average correlations across the ESGs considered. The choice of *q* is a tuneable parameter which could be chosen individually for each cancer type according to the suspected influences of the TME on the ESG signal, which are expected to vary between cancer types. In a few cases, when a particular cell type was suspected of strongly confounding the deconvolution result, this procedure was overridden by manually assigning a weight of 1 to the suspected cell type and 0 to all others in order to maximise the priority given to this cell type in the filtering process. Following definition of these weights *W*_*i*_, each ESG *g* was assigned a score defined as SC_*g*_ = -∑_*i*_ *ρ*_*g*,*i*_ ∙ *W*_*i*_. An ESG will thus have a low score if it correlates highly with many cell type marker genes, indicating that it may primarily reflect other cell types besides cancer cells and CAFs. Conversely, genes which correlate with few marker genes are given a high score. We order the ESGs by these scores and take the top *n*. This *n* is a further tuneable parameter which may be increased or decreased to reflect greater or lesser leniency towards potential confounders, which is also expected to vary between cancer types. Due to their particular interest, any of the classical EMT markers *SNAI1/2*, *TWIST1*, *VIM*, *ZEB1/2* that were removed during this process were manually added back into the filtered ESG list.

Relative expression was then defined for the ESGs as follows. For each sample *j*, we computed the sum *S*_*j*_ = ∑_*g*_ *E*_*g*,*j*_ of the expression levels for this sample across the filtered ESG list, where *E*_*g*,*j*_ denotes the expression level of ESG *g* in sample *j*. The expression levels of each ESG *g* were then replaced with the residuals of a linear regression *g* = *αS* + *β* of *g* against the vector of sample sums *S* = (*S*_*j*_)_*j*_. The purpose of this was to reduce the influence of those ESGs which correlated highly with a large number of other ESGs, and which are thus unlikely to be highly specific to either cancer cells or fibroblasts. With these relative expression levels, a gene-gene Pearson correlation matrix was constructed and its axes ordered using the SPIN Side-To-Side algorithm^37^, the distance measure being 1 minus the correlation coefficient. This ordering was refined by computing, for each ESG *g*, the average correlation of *g* with the top 20 genes in the SPIN-ordered list, and similarly for the bottom 20. Denoting these averages (*σ*_*g*,1_, *σ*_*g*,2_), ESGs for which sign(*σ*_*g*,1_) = sign(*σ*_*g*,2_) were removed (except for *SNAI1/2*, *TWIST1*, *VIM* and *ZEB1/2*), while the remainder were re-ordered by the values max(*σ*_*g*,1_, *σ*_*g*,2_) ∙ (1, −1)[which.max(*σ*_*g*,1_, *σ*_*g*,2_)]. The final filtered ESG lists used in our analysis consisted of between 130 and 260 genes.

We next calculated the validation measures to use in annotating the ESG clusters as cancer- and CAF-derived. First, for each sample *j*, we computed centred expression levels *Eʹ*_*g*,*j*_ = *E*_*g*,*j*_ – *μ*_*j*_, where *μ*_*j*_ denotes the mean of (E_*g*__,*j*_)_*g*_. For each ESG *g*, we then computed the correlation between the vector of relative expression levels (*E'*_*g*,*j*_)_*j*_ and the vector of sample purity estimates. The values displayed in Fig. 2b and Fig. 3 are the running averages of these correlation coefficients (with window size 30). Secondly, for those cancer types for which cell lines data was available from CCLE, we fitted a local regression model of the average expression levels of all genes in TCGA versus in CCLE bulk RNA-seq data, using the *loess* function in R with degree = 1, span = 0.25 and family = ‘symmetric’. The tumours vs. cell lines comparison score for each gene was then defined as the predicted value for this gene according to this model minus the expression level observed in the TCGA data. These scores were then centred and divided by the maximum absolute value of their running averages (with window size 30), which are thus bounded by −1 and 1. These running averages are displayed in the relevant figures. Thirdly, for each cancer type with an available scRNA-seq dataset, we computed the relative expression of each ESG in individual cancer cells and CAFs as follows. We calculated the average expression of each ESG separately in cancer cells and in CAFs, and centred each gene’s expression vector relative to the average of these two values. We next centred each cell’s ESGs expression vector and computed its running average with window size 30. These running averages are displayed for all cancer cells and CAFs in the relevant figures. The pEMT and CAF clusters were annotated based on the combined evidence from these validation measures. HNSC Atypical and LUSC Basal were excluded because the purity correlations exhibited the opposite pattern from the other two measures. HNSC Classical was retained despite weak support from the pattern of purity correlations, because of the strong agreement with scRNA-seq data, which we consider the most reliable measure. A similarly weak pattern of purity correlations was observed in ESCA Squamous, which was excluded due to the absence of an additional validation by scRNA-seq.

### Robustness of the deconvolution results

To identify cases where, despite our strict filtering method for the ESGs, there remained evidence of confounding by other cell types, we estimated the association of the identified pEMT and CAF clusters with these cell types using the marker gene lists used earlier. For each ESG *g* and each cell type *C*_*i*_, we computed the average correlation *ρ*_*g*,*i*_ of *g* with marker genes for *C*_*i*_, as before. We then constructed a linear regression model *ρ*_*g*,*i*_ = *α*_*i*_*r*_*g*_ + *β*_*i*_ for each *C*_*i*_, where *r*_*g*_ is the position of gene *g* in the SPIN-ordered ESG list, scaled to the range [0, 1]. Simply speaking, this model measures the change in correlation with *C*_*i*_ over the ordered ESG list. We conservatively excluded cancer types/subtypes for which the minimum regression slope min_*i*_(*α*_*i*_) was less than or equal to −0.1, indicating a change in correlation of at least 0.1 from the CAF cluster to the pEMT cluster for at least one cell type.

To quantify the overall strength of the separation into pEMT and CAF clusters, we defined within-cluster correlation by the average of the correlations for the top 30 genes in each cluster (excluding the correlation of each gene with itself), and between-cluster correlation by the average correlation of the top 30 pEMT genes with the top 30 CAF genes. To quantify the agreement of the separation with the available validation measures, we defined the between-cluster difference for a given measure by the difference between its average values for the top and bottom third of genes in the ordered gene list. The differences for each measure were divided by the mean across cancer types to aid comparison.

### Association of ESGs with pEMT and CAFs across cancer types

We defined a global ESG list by taking the union of the cancer-type-specific filtered ESG lists across those cancer types/subtypes which survived the quality control process. We assigned scores to each of these genes as follows. Firstly, for each cancer type/subtype, we transformed the expression data for the global ESG list using linear regression against the sample sums for the cancer-type-specific ESG list, as described earlier. The score for each ESG was then defined by the difference between its average correlations (in the transformed space) with the top 20 genes in the pEMT and CAF clusters. These scores were then divided by 3 times their standard deviation (across all genes, per cancer type/subtype). Any of the resulting scores which were greater than 1, respectively less than −1, were compressed to 1, resp. −1, for display in the heatmaps in Fig. 4c and Fig. S11. The significance of the association of each ESG with the pEMT and CAF clusters was measured as a *p* value computed via a t-test on the distribution of scores for that gene.

We further defined a rank for each gene *g* in the global ESG list to reflect its average position in the ordered cancer-type-specific ESG lists, giving lower weight to genes which rarely appeared in these lists. For each cancer type, if *g* was present in the cancer-type-specific ESG list, the rank of *g* was defined as its position in the ordered list divided by the total number of genes in this list. If it was not present, it was assigned a rank 0.5. For each ESG, we then calculated the 25^th^ percentile of its ranks. We defined the top *n* most common pEMT genes as the *n* ESGs with the highest such percentiles. The top *n* most common CAF genes were defined similarly, using the 75^th^ percentile.

Cancer types were clustered by first computing pairwise correlations between cancer types based on the pEMT-CAF scores for the 100 most common pEMT genes, then applying hierarchical clustering with average linkage to the resulting correlation matrix. We then cut the dendrogram into three clusters, computed each cancer type’s silhouette with respect to these three clusters, and defined as intermediates those cancer types whose silhouette was less than or equal to 0.2. Removing these intermediate cancer types resulted in three distinct clusters {*S*_*i*_}_*i* = 1,2,3_. ESGs characterising cluster *S*_*i*_ were chosen by ranking the ESGs by the difference between their average scores among cancer types/subtypes in *S*_*i*_ and those not in *S*_*i*_. To compare the pEMT clusters with overall differences between cancer types, we defined the global similarity between cancer types as follows. We computed the mean and the variance of each gene in the TCGA bulk RNA-seq dataset for each cancer type and identified the 5,000 genes with highest variance. For each pair of cancer types, we took the intersection of their respective sets of highly variable genes and calculated the correlation between their mean expression levels. These correlation values are shown in Fig. S12.

### Correlation of pEMT and CAFs with TCGA clinical annotations

We included 7 clinical features in this analysis, and for each one, we partitioned the tumours into two discrete groups having worse or better prognosis, respectively. We then used a Wilcoxon rank-sum test to compare signature scores (computed as in scRNA-seq data, with tumours taking the place of individual cells—see above) for the top 20 pEMT genes, and likewise the top 20 CAF genes, between tumours of each of these two groups. The comparisons for the examined clinical features were as follows.

- Lymph node metastasis: tumours with at least 2 metastatic lymph nodes versus those with none.
- N stage: tumours with N stage 2 or 3 versus those with N stage 0 or 1.
- Lymphovascular invasion: tumours in which lymphovascular invasion was present versus those in which it was absent.
- Grade: tumours with grade 3 or 4, or labelled “high grade”, versus those with borderline grade, grade 1 or 2, or labelled “low grade”.
- T stage: tumours with T stage 3 or 4 versus those with T stage 0, 1 or 2.
- Reduced survival: tumours with associated number of days to death lower than the 40^th^ percentile versus those with days to death higher than the 60^th^ percentile.
- Therapy resistance: tumours which recurred after follow-up treatment versus those showing complete remission/response after follow-up treatment.

Cancer types/subtypes having fewer than 10 annotated samples for a given clinical feature were excluded from analysis of that feature. In particular, the LUSC Secretory subtype is absent from this analysis as there were too few annotated samples for all examined features. We initially included M stage in our analysis, but since most cancer types/subtypes had fewer than 10 samples annotated for M stage, and since those few with 10 or more showed no significant correlations with this feature, it was excluded from later analyses. Significance thresholds to control the false discovery rate (FDR) at 0.05 were computed according to the Benjamini-Hochberg procedure, either per feature in the case of Fig. 5b or across all features for Fig. S13a.

To further examine the observed negative correlation of pEMT with lymph node metastasis in HNSC Classical tumours, we computed the pairwise correlations between these tumours across the 2000 most variable genes in the TCGA data, and ordered the resulting correlation matrix via the SPIN side-to-side algorithm^37^. The tumours were then scored as above for the pEMT program (using the inferred pEMT signature for this cancer subtype) and for an oxidative phosphorylation (OXPHOS) signature. The OXPHOS signature was identified using a Gene Set Enrichment Analysis (GSEA) test comparing HNSC Classical tumours with at least 2 lymph node metastases to those with none, using the R package clusterProfiler^57^. For visualisation purposes, the pEMT and OXPHOS scores were divided by 2 times their respective standard deviations.

### Reporting summary

Further information on research design is available in the Nature Research Reporting Summary linked to this article.

## Supplementary information

Supplementary Information

Reporting Summary

## Data Availability

This study involved re-analysis of published datasets, including scRNA-seq datasets available through the original studies, as described in Table S1, and bulk tumour datasets from TCGA, available at http://gdac.broadinstitute.org/. The breast and ovarian cancer datasets of Qian et al.^30^ are available in the ArrayExpress database under accession code E-MTAB-8107. The colorectal cancer dataset of Lee et al.^33^ is available in the NCBI Gene Expression Omnibus (GEO) database under accession code GSE132465. The head and neck cancer (HNSCC) dataset of Puram et al.^13^ is available in the GEO database under accession code GSE103322. The liver cancer dataset of Ma et al.^26^ is available in the GEO database under accession code GSE125449. The lung adenocarcinoma dataset of Kim et al.^28^ is available in the GEO database under accession code GSE131907. The lung cancer dataset of Qian et al.^30^ is available in the ArrayExpress database under accession codes E-MTAB-6149 and E-MTAB-6653. The pancreatic cancer dataset of Peng et al.^29^ is available in the Genome Sequence Archive database under accession code CRA001160. The TCGA datasets were downloaded from http://gdac.broadinstitute.org/. For each cancer type, the corresponding bulk RNA-seq dataset was downloaded by selecting the ‘illuminahiseq_rnaseqv2-RSEM_genes’ link, and the clinical dataset by selecting ‘Clinical_Pick_Tier1’. The MSigDB Hallmark EMT gene set is available on the GSEA-MSigDB website [http://www.gsea-msigdb.org/gsea/msigdb/cards/HALLMARK_EPITHELIAL_MESENCHYMAL_TRANSITION.html]. The remaining data are available within the Article, Supplementary Information or available from the authors upon request. Source data are provided with this paper.

## Source data

Source Data

## Code availability

The source code for all analyses in this study is available in a Github repository^58^, 10.5281/zenodo.4553528, URL: https://github.com/m20ty/decoupling_emt_stroma.
