# Supplementary material for: Decoupling epithelial-mesenchymal transitions from stromal profiles by integrative expression analysis
Source: Nat Commun. 2021 May 10;12:2592. doi: 10.1038/s41467-021-22800-1 (PMC8110844; doi:10.1038/s41467-021-22800-1)
Supplement: Supplementary file 1 — Supplementary Information [file 41467_2021_22800_MOESM1_ESM.pdf]

Supplementary Material

Decoupling epithelial-mesenchymal transitions from stromal profiles by integrative expression analysis

Cancer type	TCGA disease code	Source	scRNA-seq platform	Number of patients	Number of cells
Breast Cancer	BRCA	Qian et al.[1]	10x Chromium v2	14	44024
Colorectal Cancer	COADREAD	Lee et al.[2] (SMC cohort)	10x Chromium v2	23	47285
Head and Neck Squamous Cell Carcinoma (HNSCC)	HNSC	Puram et al.[3]	Smart-seq2	18	5902
Hepatocellular Carcinoma (HCC)/Intrahepatic Cholangiocarcinoma (iCCA)	LIHC	Ma et al.[4]	10x Chromium v2	19	9946
Lung Adenocarcinoma	LUAD	Kim et al.[5]	10x Chromium v2	15	57222
Lung Squamous Cell Carcinoma	LUSC	Qian et al.[1]	10x Chromium v1/v2	3	14021
Ovarian Cancer	OV	Qian et al.[1]	10x Chromium v2	5	34469
Pancreatic Ductal Adenocarcinoma (PDAC)	PAAD	Peng et al.[6]	10x Chromium v2	24	41986

Table S1. Summary of scRNA-seq datasets used in this study. The numbers of patients and cells refer only to those patients from which primary tumour samples were collected. The number of cells is the number that passed the initial quality control measures used by the authors of those studies. Both numbers represent the data prior to our own preprocessing and filtering. The lung cancer dataset of Qian et al.[1] has 8 samples in total: all of these were included in our analysis identifying malignant cells and non-malignant cell types, but only the three LUSC samples described here were used in analysis of ESGs.

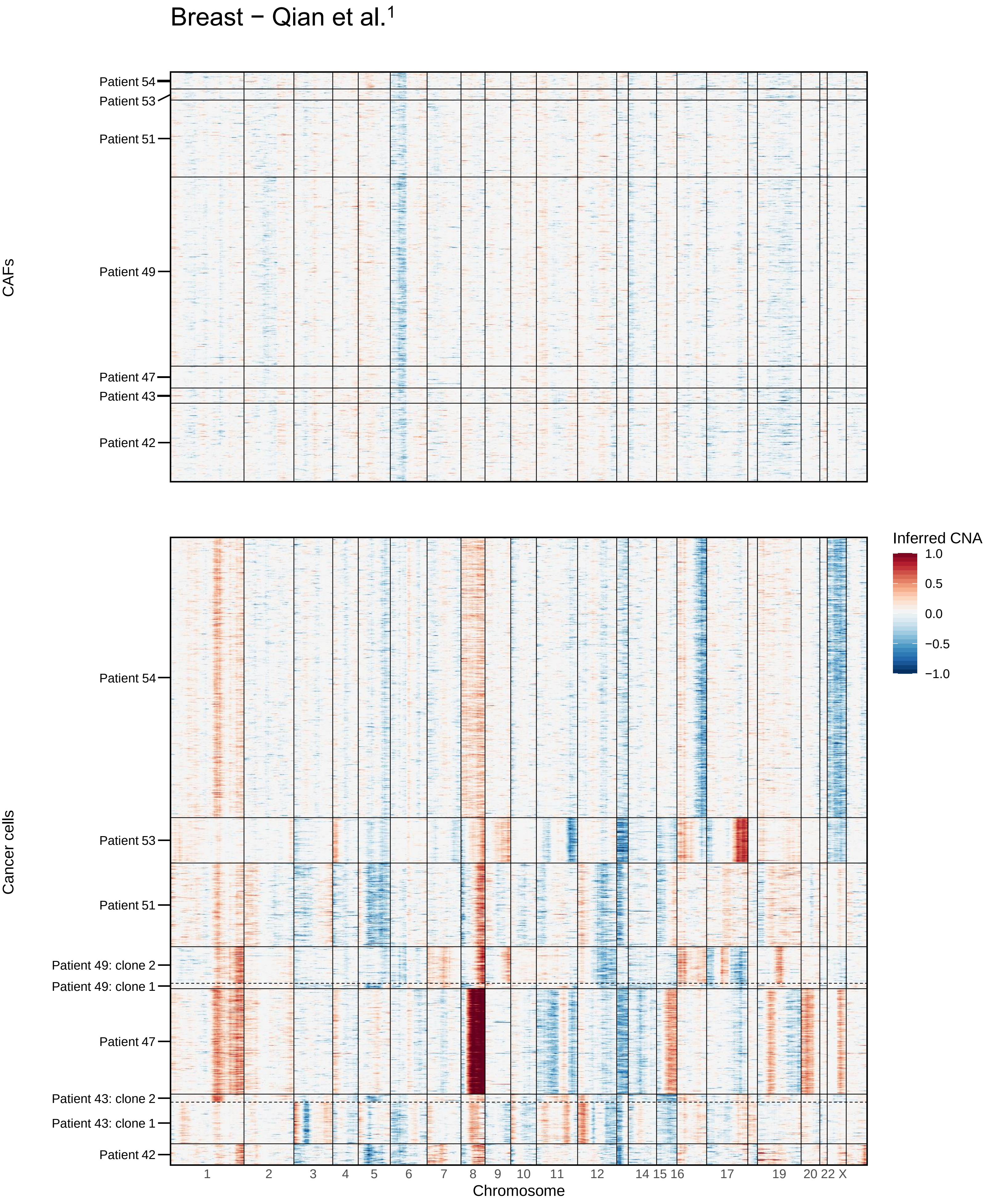

Breast − Qian et al.1
CAFs
Patient 54
Patient 53
Patient 51
Patient 49
Patient 47
Patient 43
Patient 42
Cancer cells
Patient 54
Patient 53
Patient 51
Patient 49: clone 2
Patient 49: clone 1
Patient 47
Patient 43: clone 2
Patient 43: clone 1
Patient 42
1 2 3 4 5 6 7 8 9 10 11 12 14 15 16 17 19 20 22 X
Chromosome
Inferred CNA
1.0
0.5
0.0
−0.5
−1.0

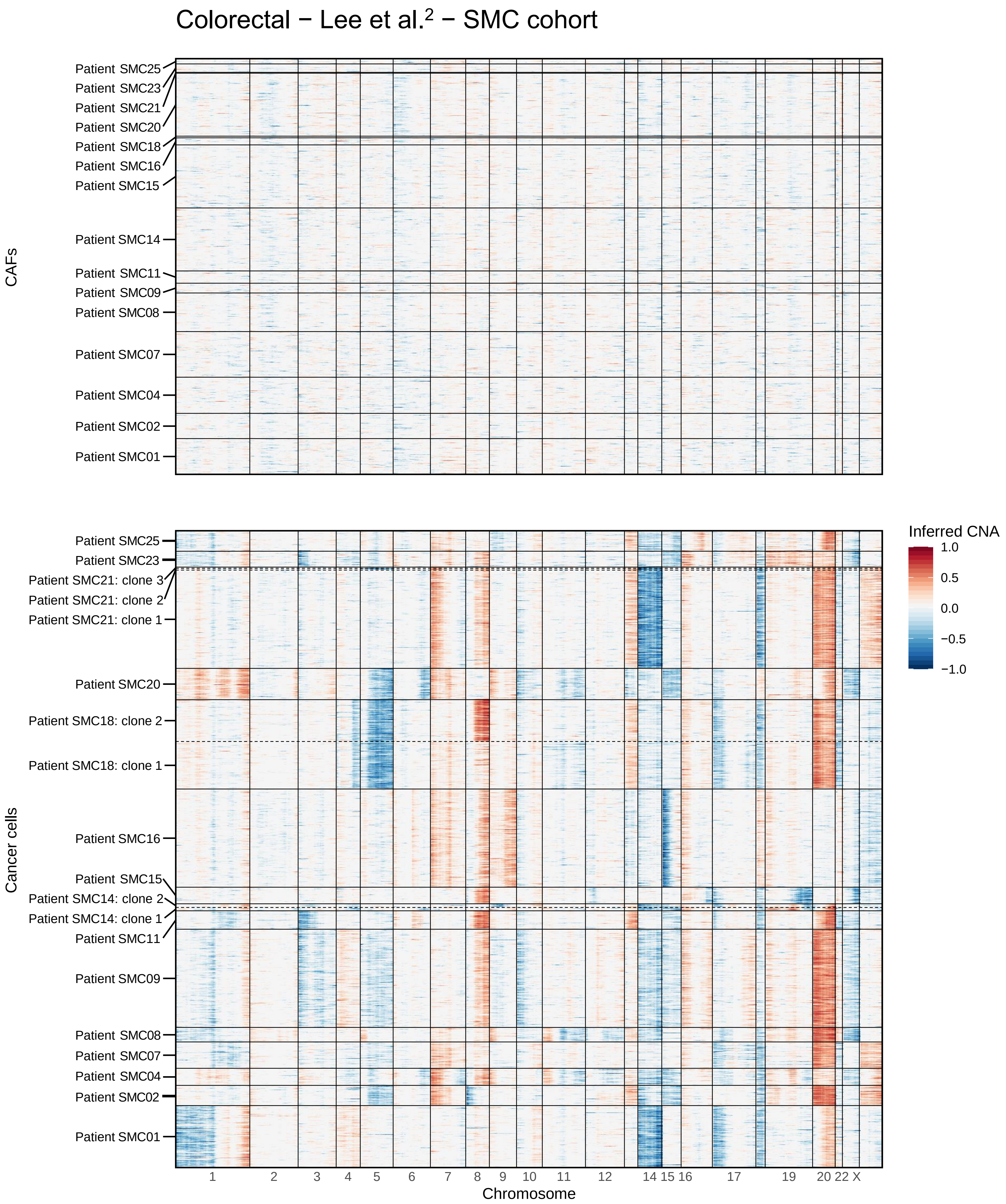

Colorectal − Lee et al.2 − SMC cohort
CAFs
Cancer cells
Patient SMC25
Patient SMC23
Patient SMC21
Patient SMC20
Patient SMC18
Patient SMC16
Patient SMC15
Patient SMC14
Patient SMC11
Patient SMC09
Patient SMC08
Patient SMC07
Patient SMC04
Patient SMC02
Patient SMC01
Patient SMC21: clone 3
Patient SMC21: clone 2
Patient SMC21: clone 1
Patient SMC18: clone 2
Patient SMC18: clone 1
Patient SMC14: clone 2
Patient SMC14: clone 1
Inferred CNA
1.0
0.5
0.0
−0.5
−1.0
1 2 3 4 5 6 7 8 9 10 11 12 14 15 16 17 19 20 22 X
Chromosome

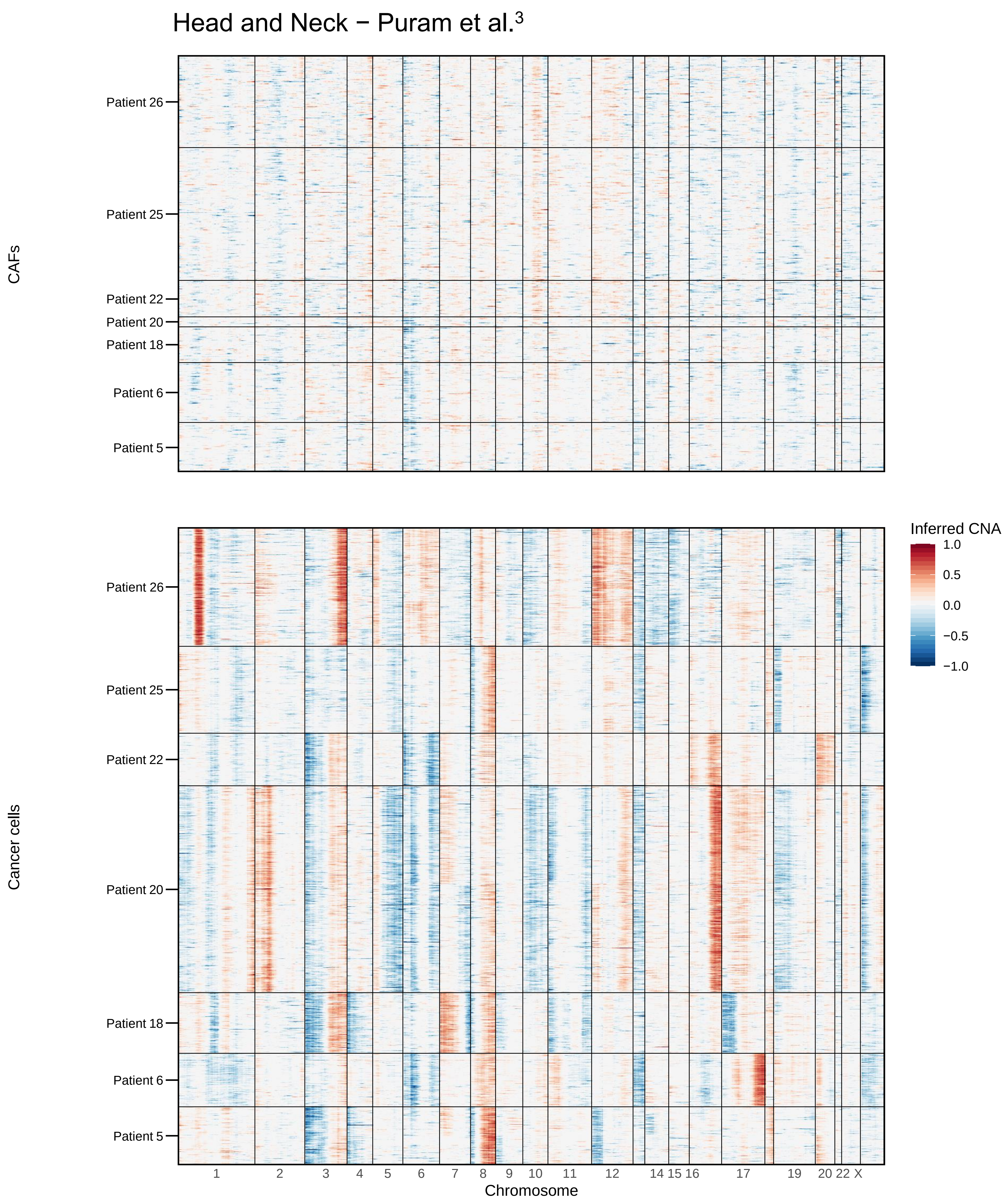

Head and Neck − Puram et al.3
CAFs
Cancer cells
Patient 26
Patient 25
Patient 22
Patient 20
Patient 18
Patient 6
Patient 5
Inferred CNA
1.0
0.5
0.0
−0.5
−1.0
1 2 3 4 5 6 7 8 9 10 11 12 14 15 16 17 19 20 22 X
Chromosome

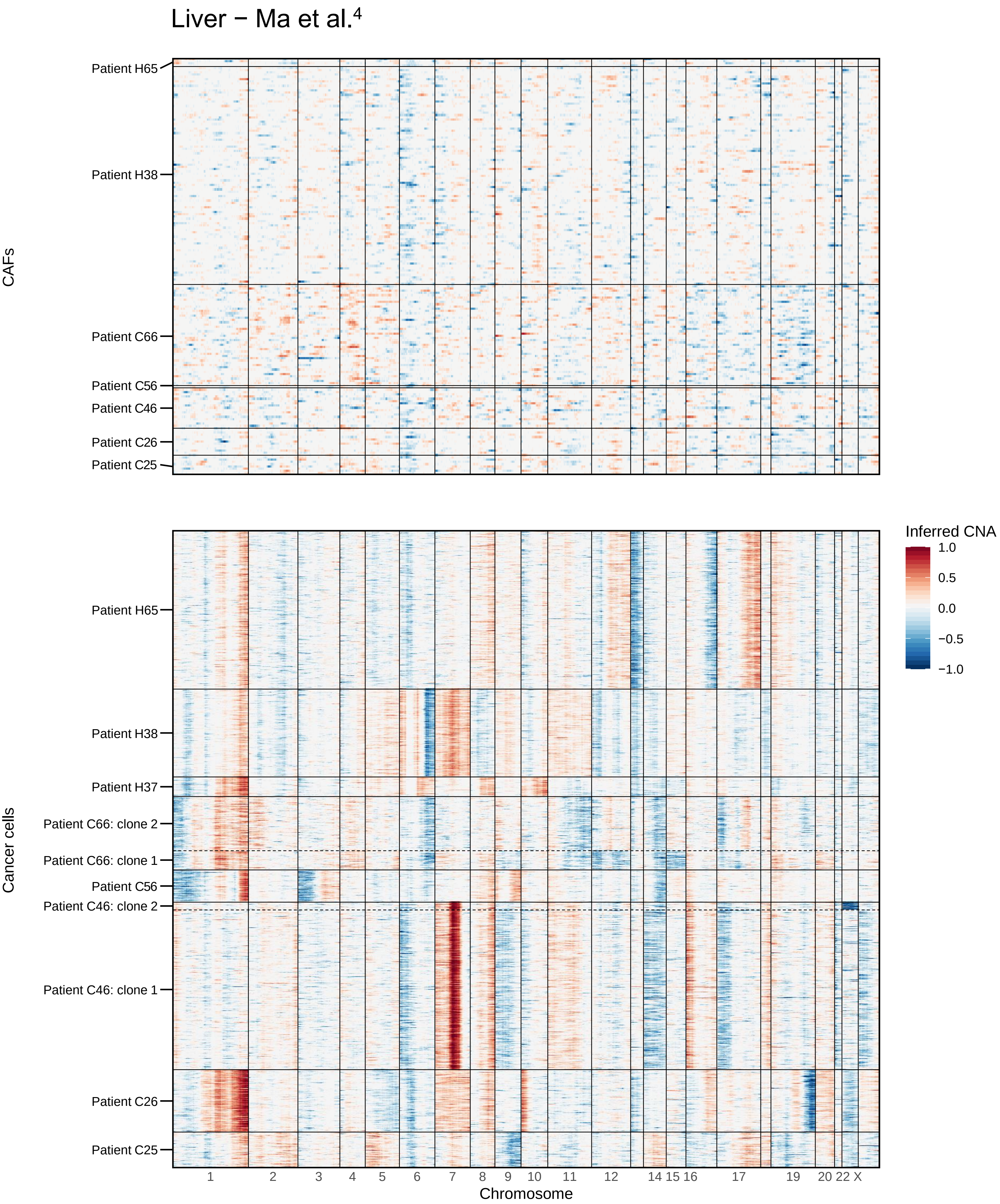

Liver − Ma et al.4
CAFs
Patient H65
Patient H38
Patient C66
Patient C56
Patient C46
Patient C26
Patient C25
Cancer cells
Patient H65
Patient H38
Patient H37
Patient C66: clone 2
Patient C66: clone 1
Patient C56
Patient C46: clone 2
Patient C46: clone 1
Patient C26
Patient C25
Inferred CNA
1.0
0.5
0.0
−0.5
−1.0
1 2 3 4 5 6 7 8 9 10 11 12 14 15 16 17 19 20 22 X
Chromosome

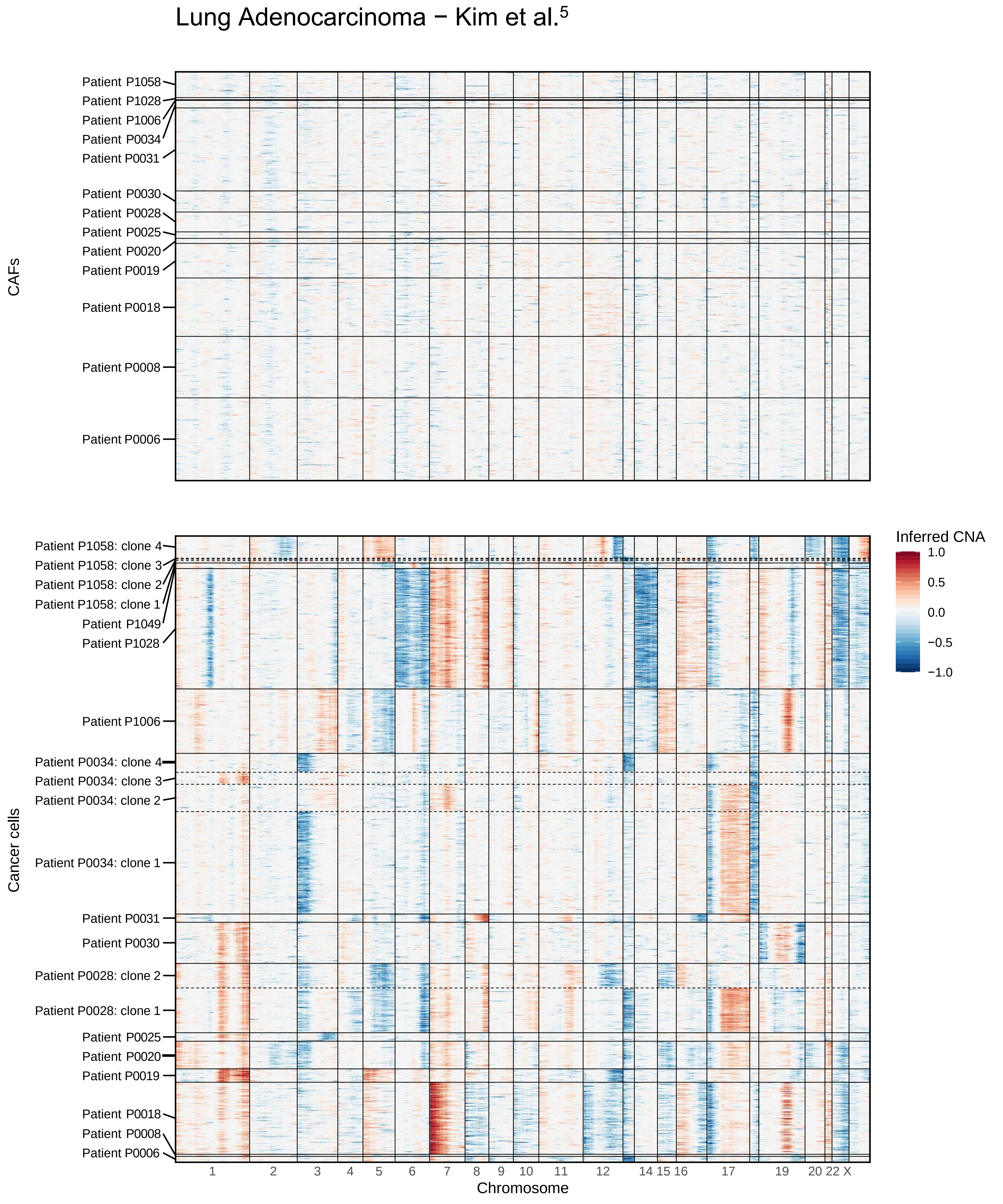

Lung Adenocarcinoma − Kim et al.5
CAFs
Patient P1058
Patient P1028
Patient P1006
Patient P0034
Patient P0031
Patient P0030
Patient P0028
Patient P0025
Patient P0020
Patient P0019
Patient P0018
Patient P0008
Patient P0006
Cancer cells
Patient P1058: clone 4
Patient P1058: clone 3
Patient P1058: clone 2
Patient P1058: clone 1
Patient P1049
Patient P1028
Patient P1006
Patient P0034: clone 4
Patient P0034: clone 3
Patient P0034: clone 2
Patient P0034: clone 1
Patient P0031
Patient P0030
Patient P0028: clone 2
Patient P0028: clone 1
Patient P0025
Patient P0020
Patient P0019
Patient P0018
Patient P0008
Patient P0006
Inferred CNA
1.0
0.5
0.0
−0.5
−1.0
1
2
3
4
5
6
7
8
9
10
11
12
14
15
16
17
19
20
22
X
Chromosome

Lung − Qian et al.[1]

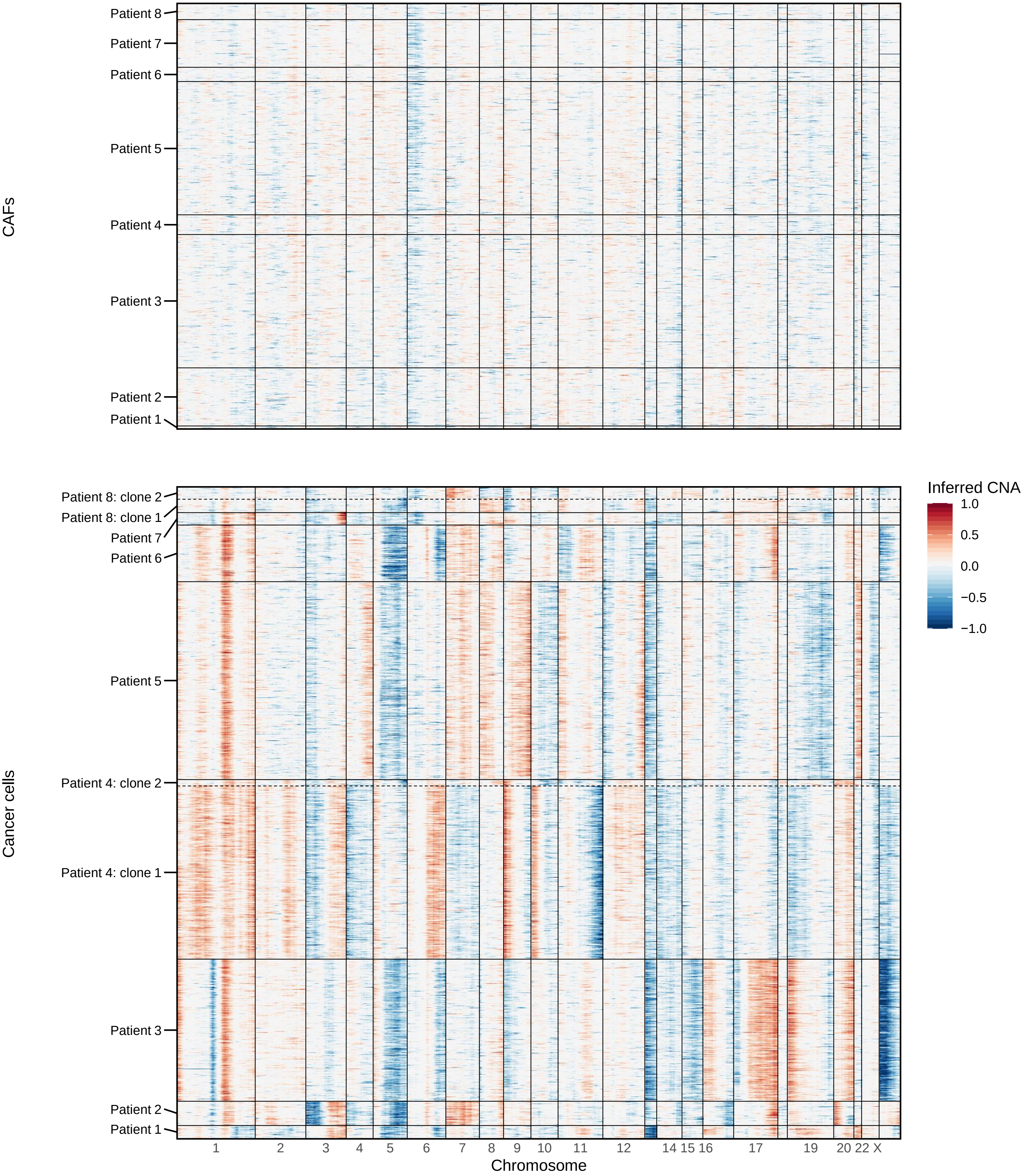

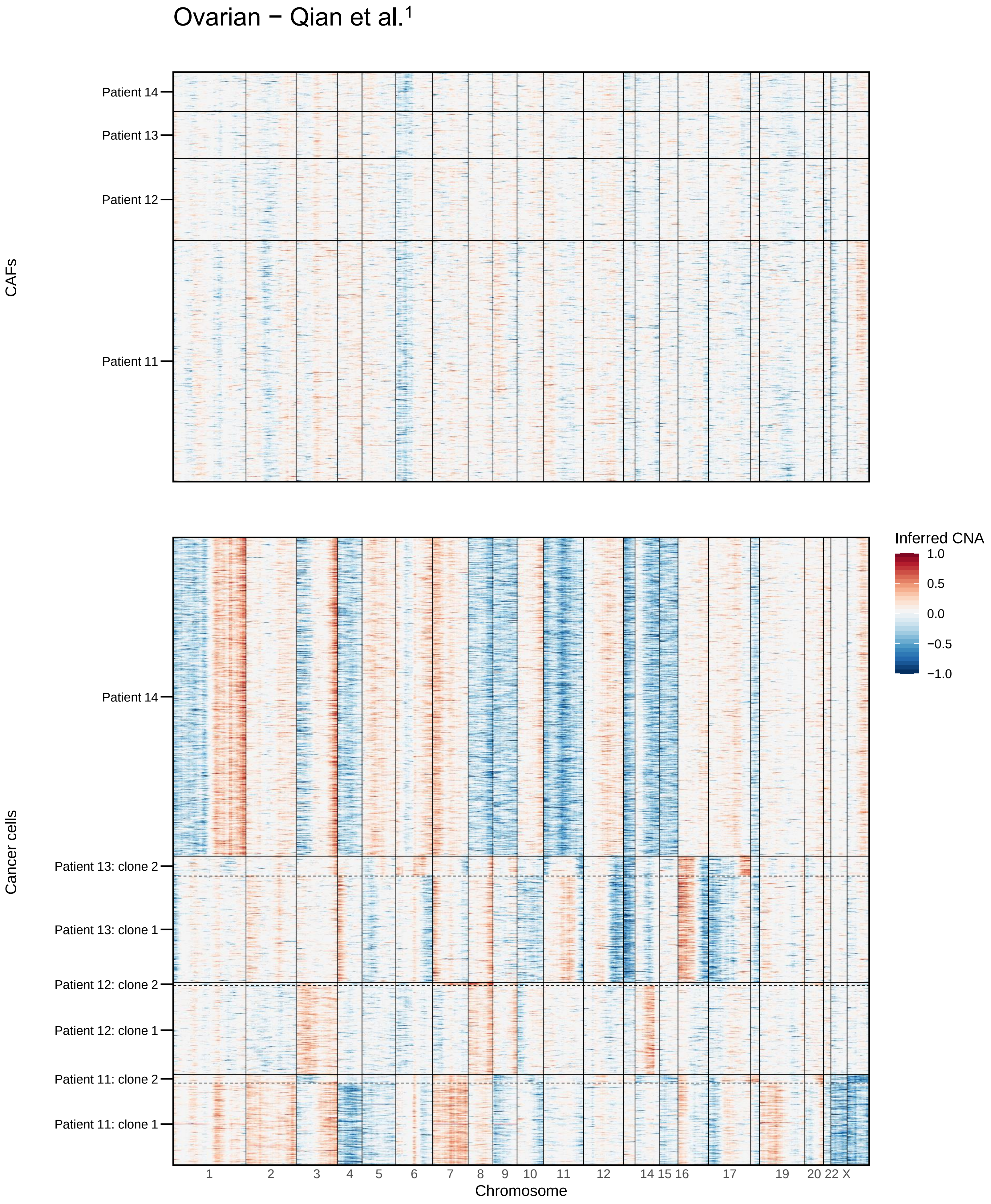
Ovarian − Qian et al.1
CAFs
Patient 14
Patient 13
Patient 12
Patient 11
Cancer cells
Patient 14
Patient 13: clone 2
Patient 13: clone 1
Patient 12: clone 2
Patient 12: clone 1
Patient 11: clone 2
Patient 11: clone 1
Inferred CNA
1.0
0.5
0.0
−0.5
−1.0
1
2
3
4
5
6
7
8
9
10
11
12
14
15
16
17
19
20
22
X
Chromosome

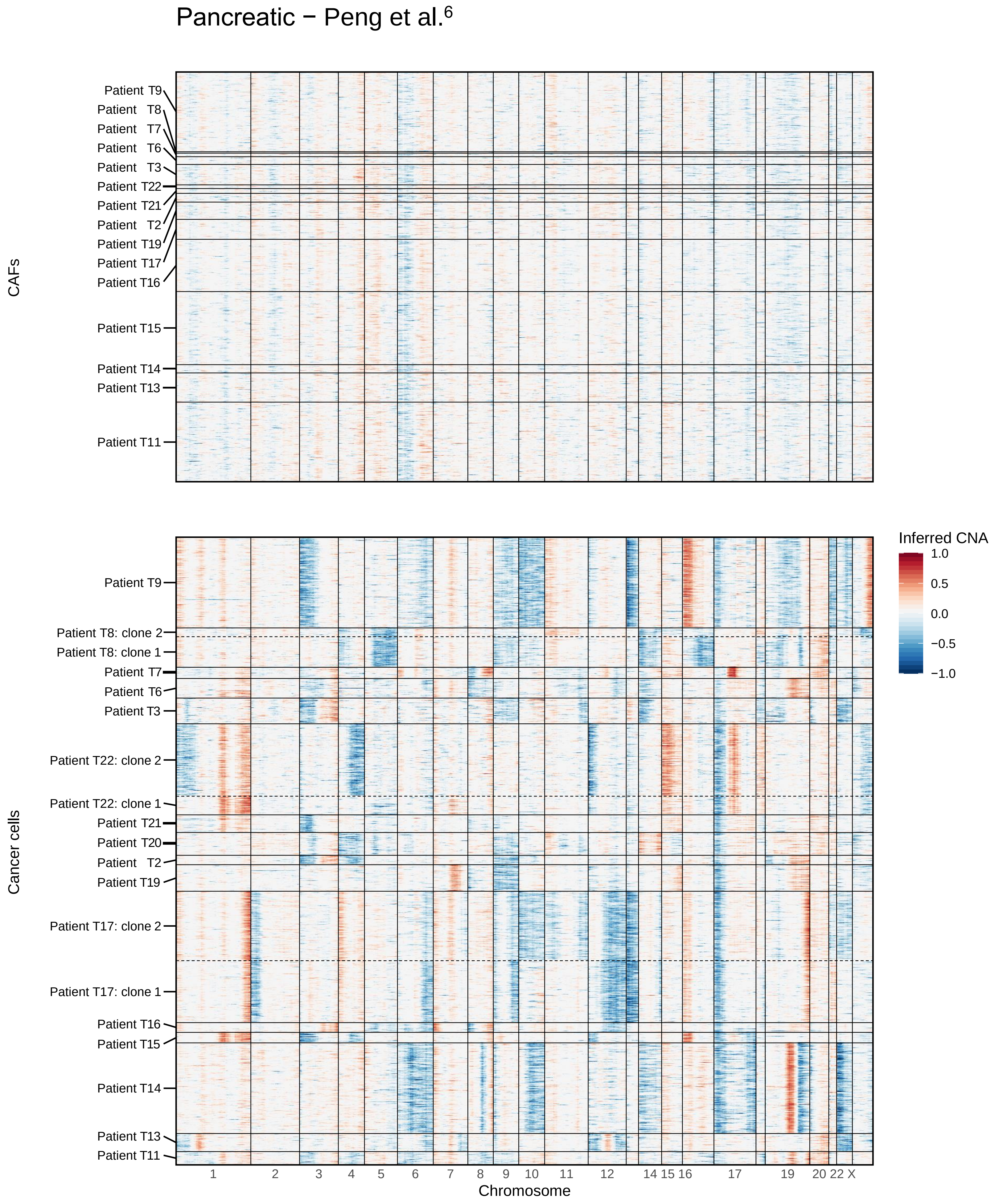

Pancreatic − Peng et al.6
CAFs
Patient T9
Patient T8
Patient T7
Patient T6
Patient T3
Patient T22
Patient T21
Patient T2
Patient T19
Patient T17
Patient T16
Patient T15
Patient T14
Patient T13
Patient T11
Cancer cells
Patient T9
Patient T8: clone 2
Patient T8: clone 1
Patient T7
Patient T6
Patient T3
Patient T22: clone 2
Patient T22: clone 1
Patient T21
Patient T20
Patient T2
Patient T19
Patient T17: clone 2
Patient T17: clone 1
Patient T16
Patient T15
Patient T14
Patient T13
Patient T11
1 2 3 4 5 6 7 8 9 10 11 12 14 15 16 17 19 20 22 X
Chromosome
Inferred CNA
1.0
0.5
0.0
−0.5
−1.0

Figure S1. Inferred copy number alterations (CNAs) in CAFs and cancer cells. Heatmaps showing the inferred CNAs in CAFs (upper panel) and cancer cells (lower panel) for each of the 8 scRNA-seq datasets examined. Rows correspond to individual cells and are grouped by patient and, where relevant, by subclone. Columns correspond to individual genes and are ordered by chromosomal location. Positive values indicate chromosomal amplification, while negative values indicate deletion. Patients or subclones having disproportionately many cells were downsampled for presentation purposes. Heatmaps for the lung cancer dataset of Qian et al.[1] include all samples from this dataset, not only the three LUSC samples. In some datasets, a weak signal of depletion of chromosome 6p is visible in the CAFs, which is likely a technical artifact relating to the choice of reference cells.

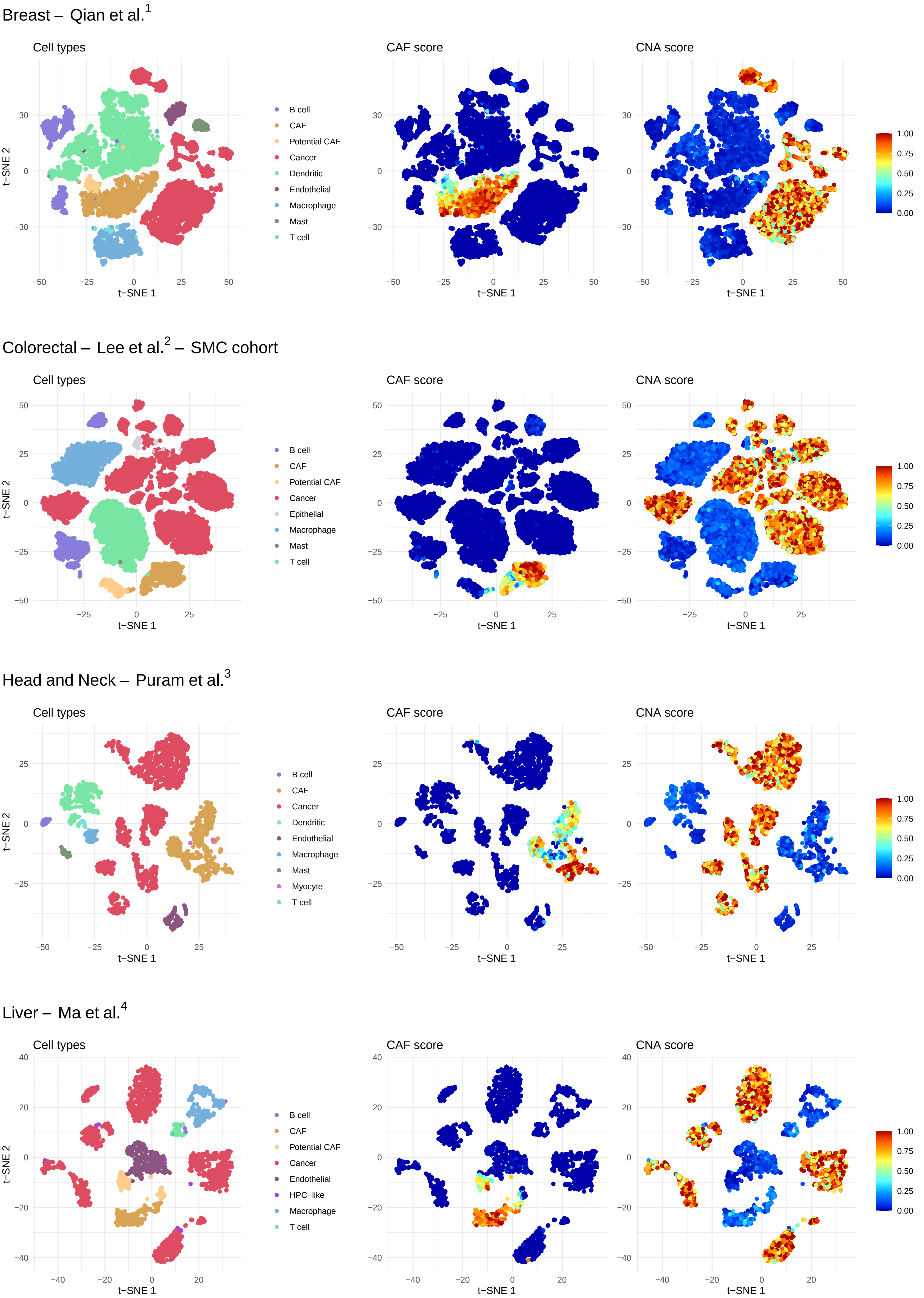

Breast – Qian et al.1
Cell types
CAF score
CNA score
B cell
CAF
Potential CAF
Cancer
Dendritic
Endothelial
Macrophage
Mast
T cell
t−SNE 1
t−SNE 2
Colorectal – Lee et al.2 – SMC cohort
Cell types
CAF score
CNA score
B cell
CAF
Potential CAF
Cancer
Epithelial
Macrophage
Mast
T cell
t−SNE 1
t−SNE 2
Head and Neck – Puram et al.3
Cell types
CAF score
CNA score
B cell
CAF
Cancer
Dendritic
Endothelial
Macrophage
Mast
Myocyte
T cell
t−SNE 1
t−SNE 2
Liver – Ma et al.4
Cell types
CAF score
CNA score
B cell
CAF
Potential CAF
Cancer
Endothelial
HPC−like
Macrophage
T cell
t−SNE 1
t−SNE 2
1.00
0.75
0.50
0.25
0.00

Lung Adenocarcinoma – Kim et al.[5]

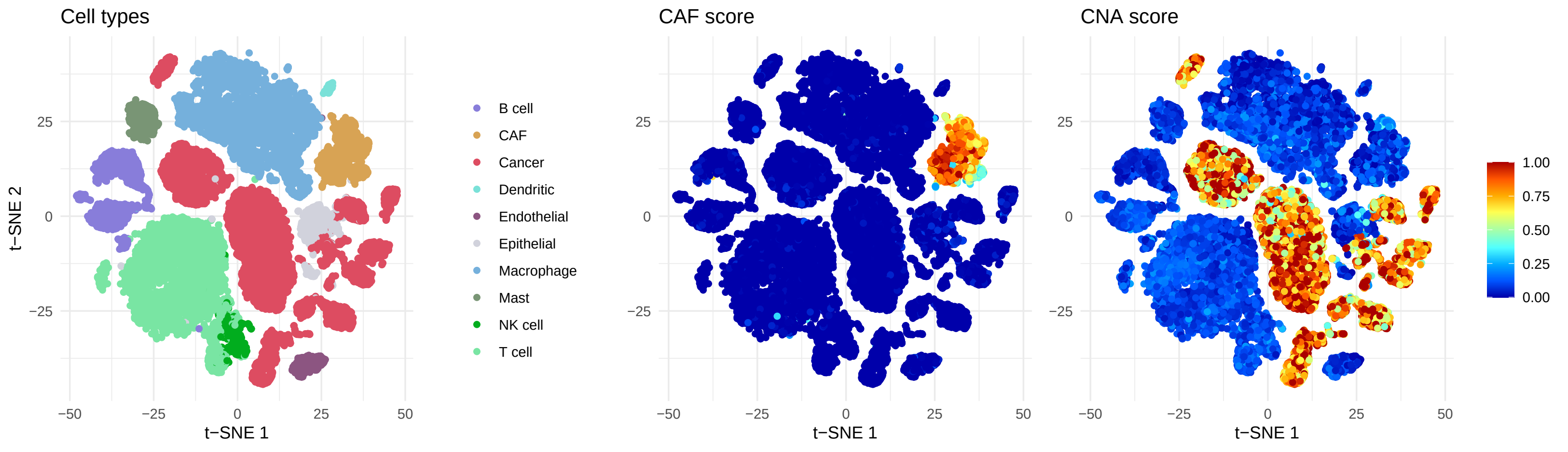

Lung – Qian et al.[1]

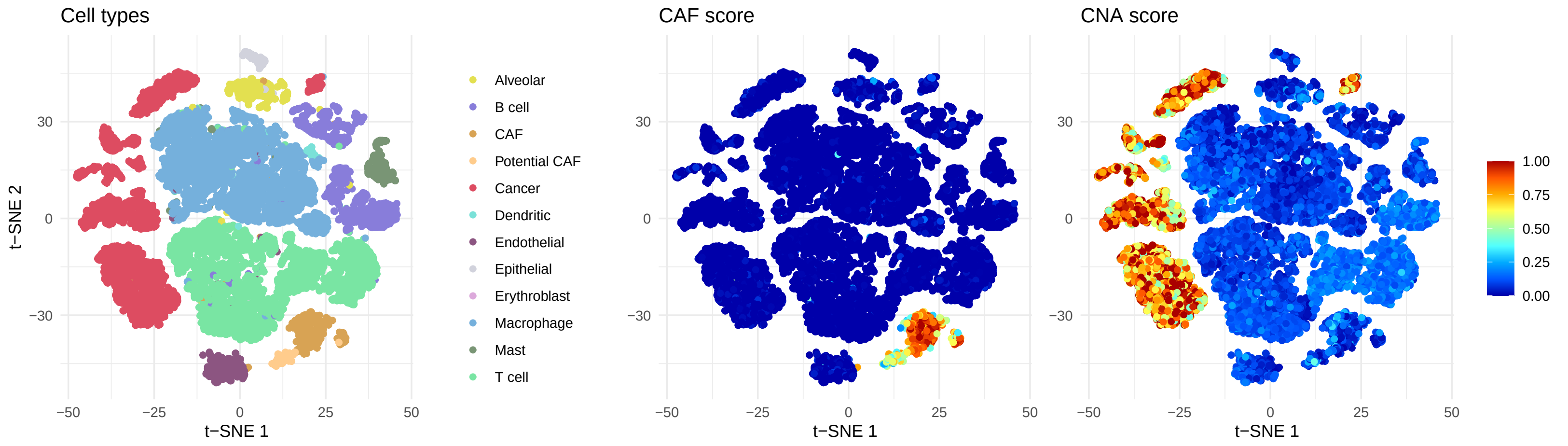

Ovarian – Qian et al.[1]

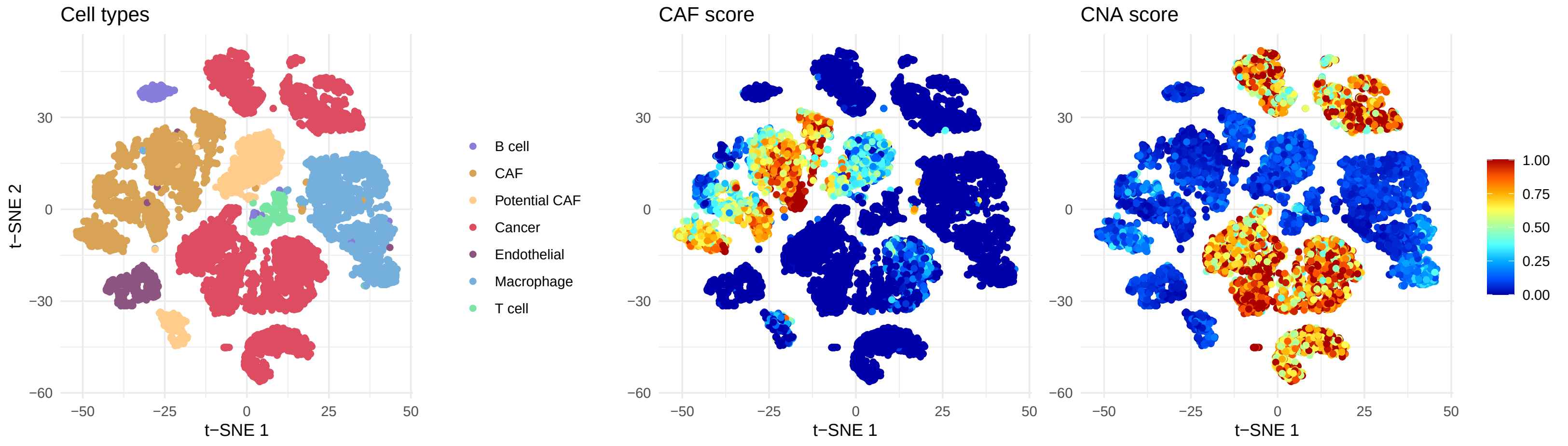

Pancreatic – Peng et al.[6]

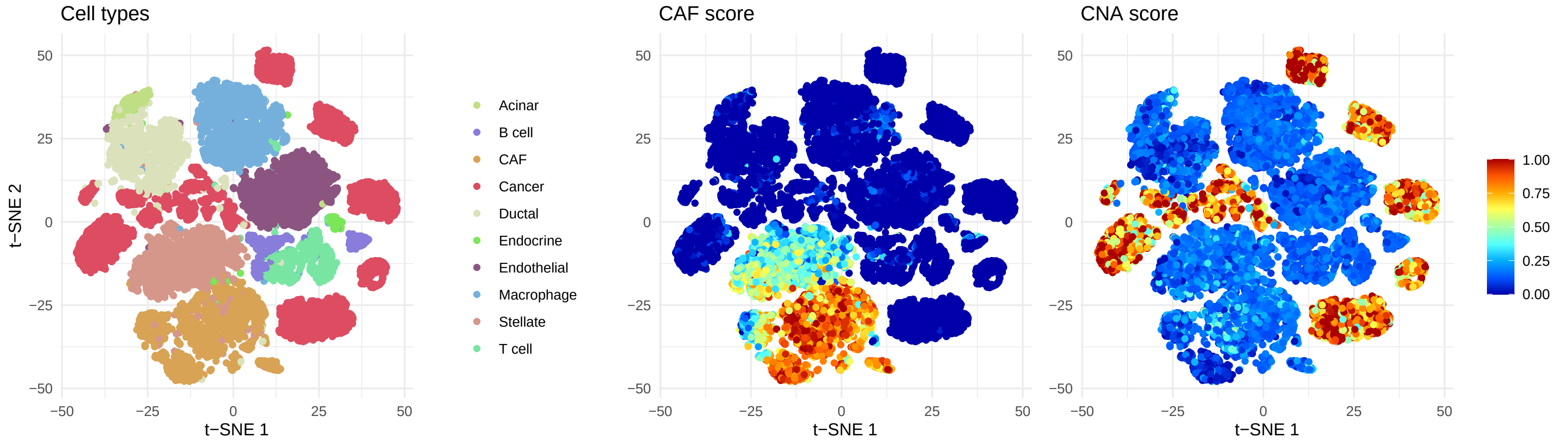

Figure S2. Summary of cell type assignments and distinction between cancer cells and CAFs. t-SNE plots for each of the 8 scRNA-seq datasets examined (rows) with points corresponding to individual cells, coloured by cell type (left panel), CAF score (middle panel) and CNA score (right panel). "Epithelial" refers to non-malignant epithelial cells; "Potential CAF" refers to CAF-like cells with ambiguous identity. In the colorectal cohort, the potential CAFs were annotated as CAFs by the authors of the corresponding study, but were found in our analysis to more closely resemble endothelial cells. Plots for the lung cancer dataset of Qian et al.[1] include all samples from this dataset, not only the three LUSC samples.

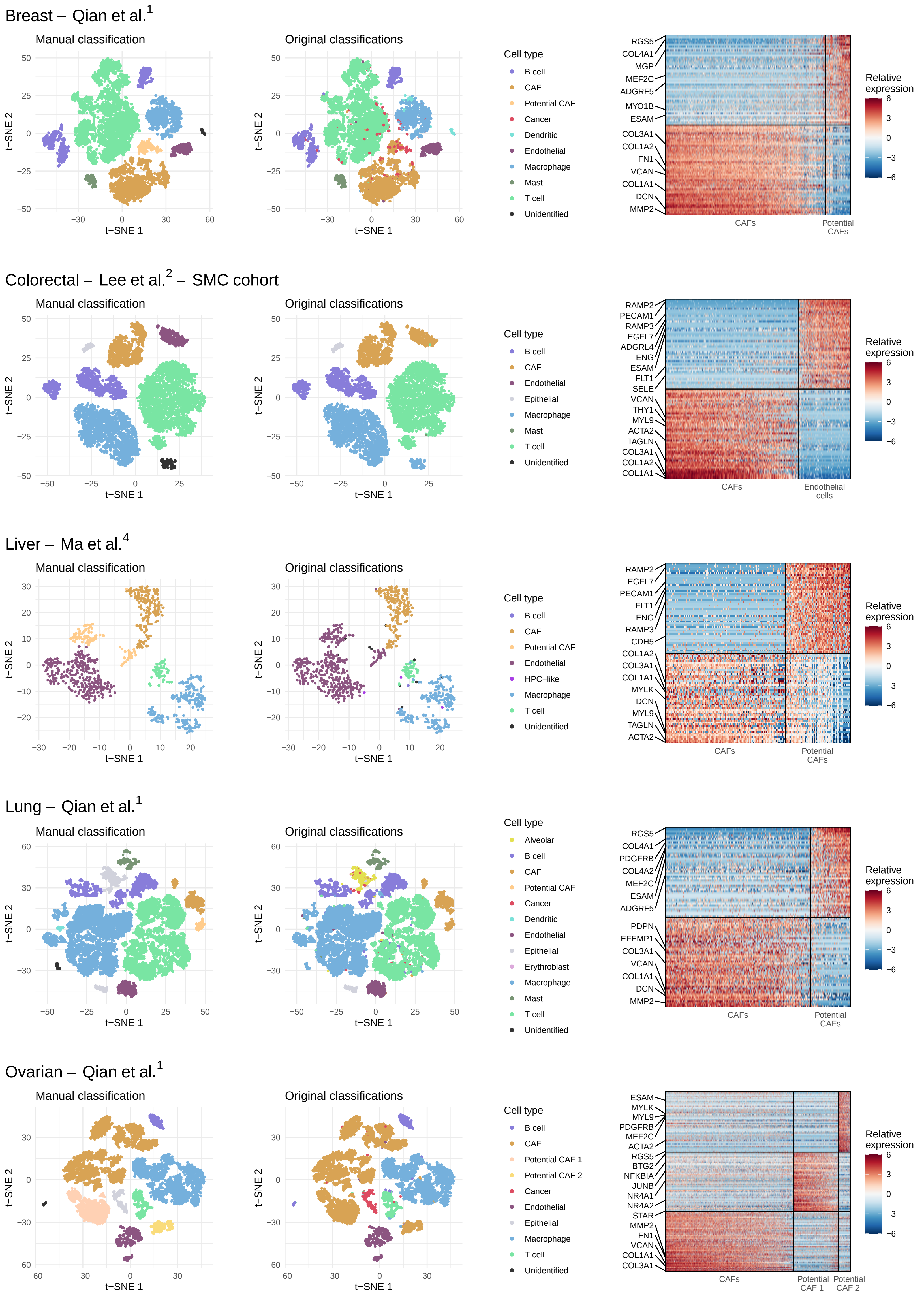
Breast – Qian et al.1
Manual classification
Original classifications
t−SNE 1
t−SNE 2
Cell type
B cell
CAF
Potential CAF
Cancer
Dendritic
Endothelial
Macrophage
Mast
T cell
Unidentified
RGS5
COL4A1
MGP
MEF2C
ADGRF5
MYO1B
ESAM
COL3A1
COL1A2
FN1
VCAN
COL1A1
DCN
MMP2
CAFs
Potential CAFs
Relative expression
Colorectal – Lee et al.2 – SMC cohort
Manual classification
Original classifications
Cell type
B cell
CAF
Endothelial
Epithelial
Macrophage
Mast
T cell
Unidentified
RAMP2
PECAM1
RAMP3
EGFL7
ADGRL4
ENG
ESAM
FLT1
SELE
VCAN
THY1
MYL9
ACTA2
TAGLN
COL3A1
COL1A2
COL1A1
CAFs
Endothelial cells
Relative expression
Liver – Ma et al.4
Manual classification
Original classifications
Cell type
B cell
CAF
Potential CAF
Endothelial
HPC−like
Macrophage
T cell
Unidentified
RAMP2
EGFL7
PECAM1
FLT1
ENG
RAMP3
CDH5
COL1A2
COL3A1
COL1A1
MYLK
DCN
MYL9
TAGLN
ACTA2
CAFs
Potential CAFs
Relative expression
Lung – Qian et al.1
Manual classification
Original classifications
Cell type
Alveolar
B cell
CAF
Potential CAF
Cancer
Dendritic
Endothelial
Epithelial
Erythroblast
Macrophage
Mast
T cell
Unidentified
RGS5
COL4A1
PDGFRB
COL4A2
MEF2C
ESAM
ADGRF5
PDPN
EFEMP1
COL3A1
VCAN
COL1A1
DCN
MMP2
CAFs
Potential CAFs
Relative expression
Ovarian – Qian et al.1
Manual classification
Original classifications
Cell type
B cell
CAF
Potential CAF 1
Potential CAF 2
Cancer
Endothelial
Epithelial
Macrophage
T cell
Unidentified
ESAM
MYLK
MYL9
PDGFRB
MEF2C
ACTA2
RGS5
BTG2
NFKBIA
JUNB
NR4A1
NR4A2
STAR
MMP2
FN1
VCAN
COL1A1
COL3A1
CAFs
Potential CAF 1
Potential CAF 2
Relative expression

Figure S3. Summary of non-malignant cell type assignments and distinction between strict and lenient CAF definitions. For each of the 8 scRNA-seq datasets examined (rows), t-SNE plots (left two columns) with points corresponding to non-malignant cells and coloured by our own cell type classifications (leftmost column) and by the classifications published with the original datasets (middle column), along with heatmaps (rightmost column) of relative expression levels of the top 50 differentially expressed genes in each cluster of CAFs and potential CAFs (or endothelial cells in the colorectal cohort). Columns of the heatmaps correspond to individual cells and rows correspond to genes, with selected genes annotated at the side. Plots for the lung cancer dataset of Qian et al.[1] include all samples from this dataset, not only the three LUSC samples.

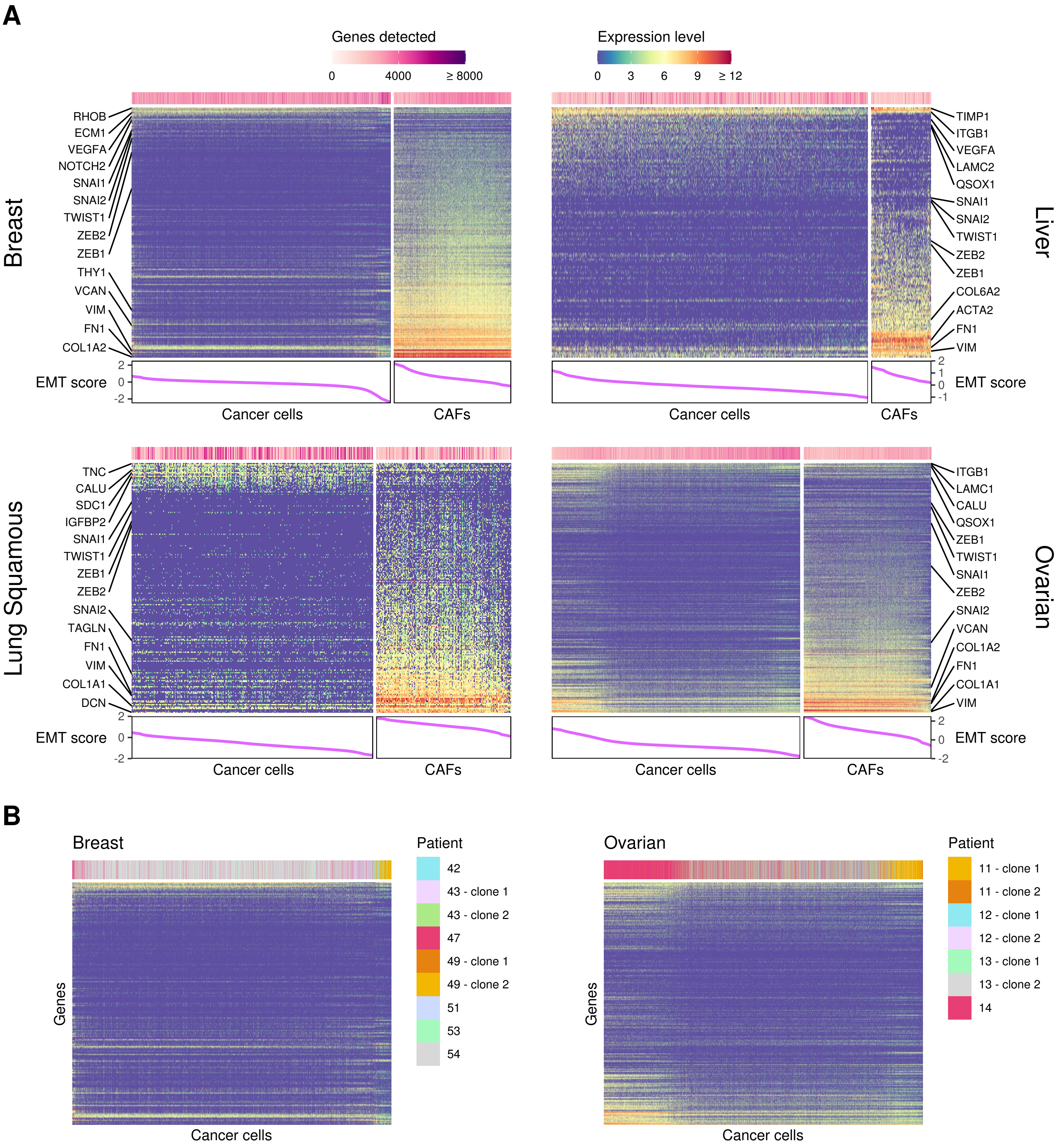

A
Genes detected
0
4000
≥ 8000
Expression level
0
3
6
9
≥ 12
Breast
RHOB
ECM1
VEGFA
NOTCH2
SNAI1
SNAI2
TWIST1
ZEB2
ZEB1
THY1
VCAN
VIM
FN1
COL1A2
EMT score
Cancer cells
CAFs
Liver
TIMP1
ITGB1
VEGFA
LAMC2
QSOX1
SNAI1
SNAI2
TWIST1
ZEB2
ZEB1
COL6A2
ACTA2
FN1
VIM
Lung Squamous
TNC
CALU
SDC1
IGFBP2
SNAI1
TWIST1
ZEB1
ZEB2
SNAI2
TAGLN
FN1
VIM
COL1A1
DCN
Ovarian
ITGB1
LAMC1
CALU
QSOX1
ZEB1
TWIST1
SNAI1
ZEB2
SNAI2
VCAN
COL1A2
FN1
COL1A1
VIM
B
Breast
Patient
42
43 - clone 1
43 - clone 2
47
49 - clone 1
49 - clone 2
51
53
54
Genes
Cancer cells
Ovarian
Patient
11 - clone 1
11 - clone 2
12 - clone 1
12 - clone 2
13 - clone 1
13 - clone 2
14
Genes
Cancer cells

Figure S4. Expression of ESGs in cancer cells and CAFs by scRNA-seq. (**A**) Heatmaps showing expression levels of ESGs (rows) in cancer cells and CAFs (columns) in the 4 scRNA-seq datasets (panels) not shown in **Fig. 1B**. Columns (cells) are ordered by their EMT scores (see **Methods**), which are shown in the line graphs below the heatmaps. The bar above each heatmap shows the number of genes detected in each cell. (**B**) Heatmaps as in (**A**) for cancer cells in the breast and ovarian cohorts, with bars above coloured by patient number and subclone.

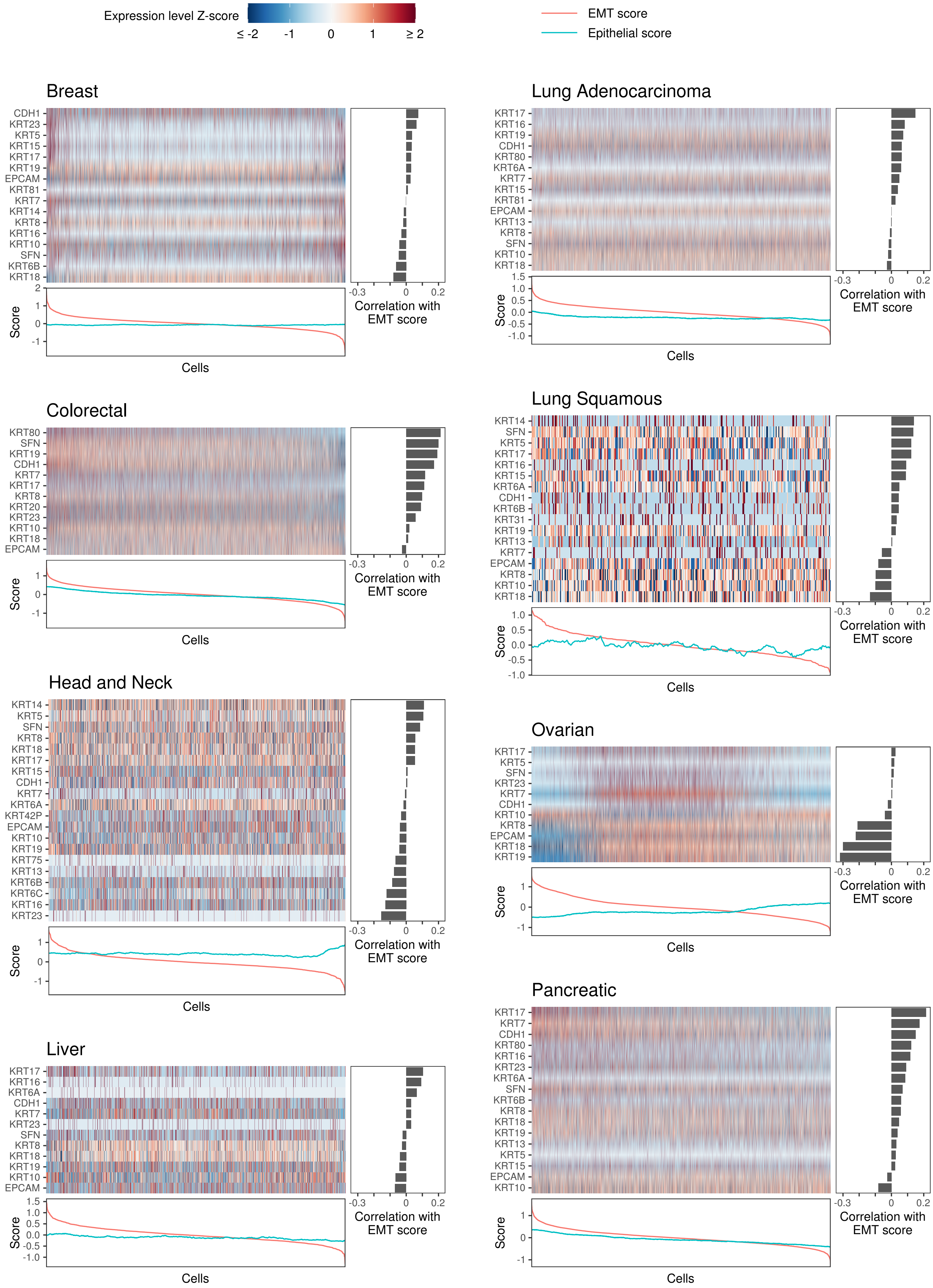
Expression level Z-score
≤ -2
-1
0
1
≥ 2
EMT score
Epithelial score
Breast
CDH1
KRT23
KRT5
KRT15
KRT17
KRT19
EPCAM
KRT81
KRT7
KRT14
KRT8
KRT16
KRT10
SFN
KRT6B
KRT18
Score
Cells
Correlation with EMT score
Lung Adenocarcinoma
KRT17
KRT16
KRT19
CDH1
KRT80
KRT6A
KRT7
KRT15
KRT81
EPCAM
KRT13
KRT8
SFN
KRT10
KRT18
Colorectal
KRT80
SFN
KRT19
CDH1
KRT7
KRT17
KRT8
KRT20
KRT23
KRT10
KRT18
EPCAM
Lung Squamous
KRT14
SFN
KRT5
KRT17
KRT16
KRT15
KRT6A
CDH1
KRT6B
KRT31
KRT19
KRT13
KRT7
EPCAM
KRT8
KRT10
KRT18
Head and Neck
KRT14
KRT5
SFN
KRT8
KRT18
KRT17
KRT15
CDH1
KRT7
KRT6A
KRT42P
EPCAM
KRT10
KRT19
KRT75
KRT13
KRT6B
KRT6C
KRT16
KRT23
Ovarian
KRT17
KRT5
SFN
KRT23
KRT7
CDH1
KRT10
KRT8
EPCAM
KRT18
KRT19
Liver
KRT17
KRT16
KRT6A
CDH1
KRT7
KRT23
SFN
KRT8
KRT18
KRT19
KRT10
EPCAM
Pancreatic
KRT17
KRT7
CDH1
KRT80
KRT16
KRT23
KRT6A
SFN
KRT6B
KRT8
KRT18
KRT19
KRT13
KRT5
KRT15
EPCAM
KRT10

Figure S5. Comparison of epithelial marker gene expression with EMT score. Heatmaps showing the relative expression levels (defined as Z scores) of epithelial marker genes (rows) in cancer cells (columns) in each of the 8 scRNA-seq datasets analysed. The epithelial markers are chosen separately for each cancer type based on having high expression levels in the corresponding dataset. The line plots beneath show the EMT and epithelial scores for these cells, and the bar plots to the right show the correlation of each epithelial gene's expression levels with the EMT scores. The cells are ordered by their EMT scores.

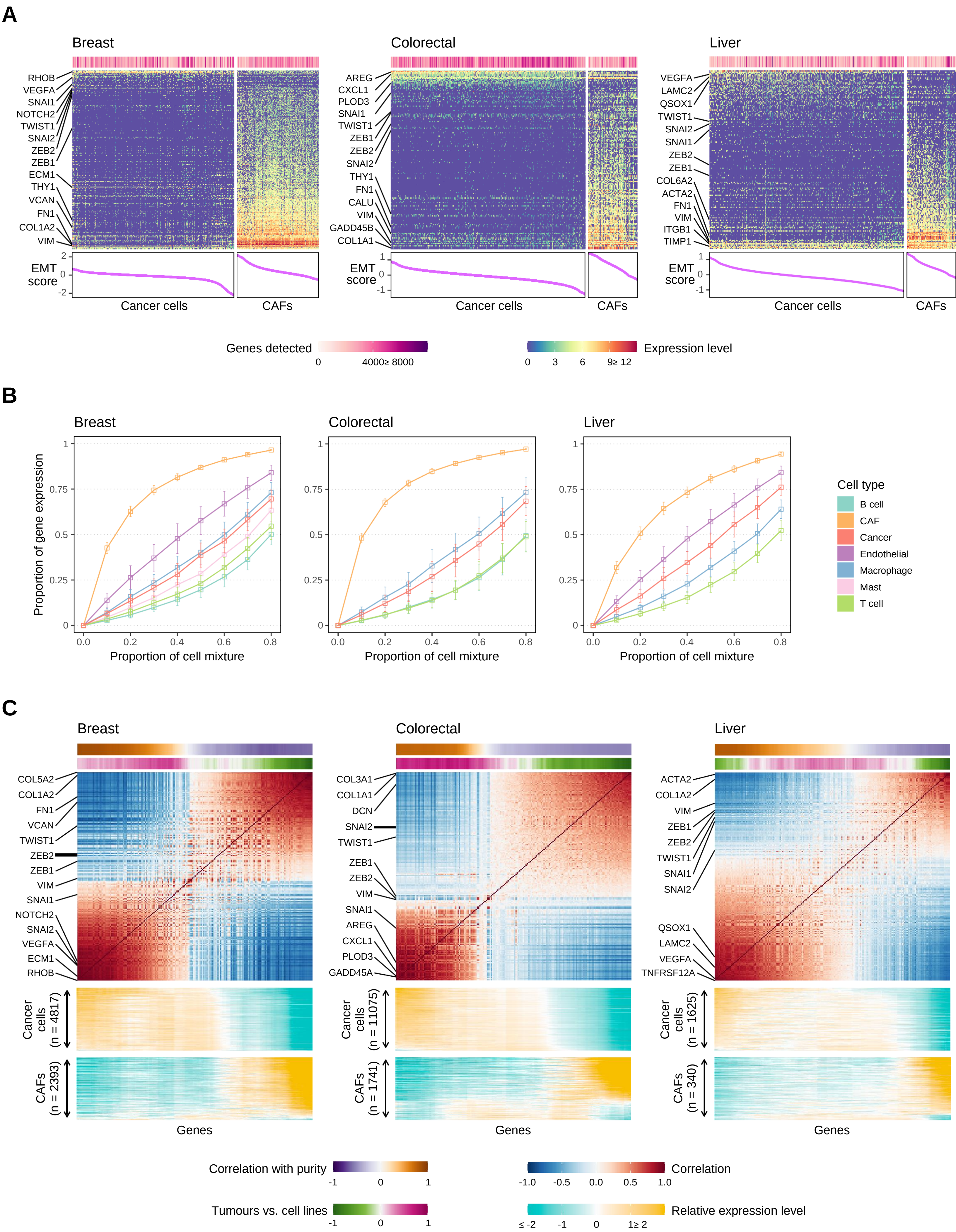
A
Breast
Colorectal
Liver
RHOB
VEGFA
SNAI1
NOTCH2
TWIST1
SNAI2
ZEB2
ZEB1
ECM1
THY1
VCAN
FN1
COL1A2
VIM
AREG
CXCL1
PLOD3
SNAI1
TWIST1
ZEB1
ZEB2
SNAI2
THY1
FN1
CALU
VIM
GADD45B
COL1A1
VEGFA
LAMC2
QSOX1
TWIST1
SNAI2
SNAI1
ZEB2
ZEB1
COL6A2
ACTA2
FN1
VIM
ITGB1
TIMP1
EMT score
Cancer cells
CAFs
Genes detected
0
4000
≥ 8000
Expression level
0
3
6
9
≥ 12
B
Proportion of gene expression
Proportion of cell mixture
Cell type
B cell
CAF
Cancer
Endothelial
Macrophage
Mast
T cell
C
COL5A2
COL1A2
FN1
VCAN
TWIST1
ZEB2
ZEB1
VIM
SNAI1
NOTCH2
SNAI2
VEGFA
ECM1
RHOB
COL3A1
COL1A1
DCN
SNAI2
TWIST1
ZEB1
ZEB2
VIM
SNAI1
AREG
CXCL1
PLOD3
GADD45A
ACTA2
COL1A2
VIM
ZEB1
ZEB2
TWIST1
SNAI1
SNAI2
QSOX1
LAMC2
VEGFA
TNFRSF12A
Cancer cells (n = 4817)
CAFs (n = 2393)
Cancer cells (n = 11075)
CAFs (n = 1741)
Cancer cells (n = 1625)
CAFs (n = 340)
Genes
Correlation with purity
Correlation
Tumours vs. cell lines
Relative expression level
≤ -2
1
≥ 2

Figure S6. Summary of scRNA-seq analysis using lenient CAF definitions. (**A**) Heatmaps showing expression levels of ESGs (rows) in cancer cells and CAFs (columns) in 3 scRNA-seq datasets, using the "lenient" definition for CAFs in each case (which includes CAFs and "potential CAFs", or endothelial cells in the colorectal cohort). Columns (cells) are ordered by their EMT scores (see **Methods**), which are shown in the line graphs below the heatmaps. The bar above each heatmap shows the number of genes detected in each cell. (**B**) Line plots showing the relative contributions of different cell types to ESG expression, for various fractions of tumour composition, in simulated bulk expression profiles based on the 3 scRNA-seq datasets shown in (**A**), using the "lenient" CAF definitions in the simulations. Each point represents the average proportion of EMT signature gene expression coming from the corresponding cell type in a collection of 100 simulated tumours with the given fraction of that cell type and varying proportions of the other cell types. Error bars show the standard deviation over the set of 100 simulations. (**C**) ESG co-expression matrices derived from simulated bulk expression data based on the 3 scRNA-seq datasets shown in (**A**), using the "lenient" CAF definitions in the simulations. ESGs are ordered by the SPIN side-to-side algorithm[7] with slight modifications (see **Methods**) and annotated with two colour-coded panels at the top: (1) correlations with simulated tumour purity (Pearson correlation coefficient); and (2) comparison of expression levels in simulated tumours versus in cell lines, where positive numbers indicate higher expression in tumours than in cell lines. Heatmaps below the co-expression matrices show the relative expression levels of ESGs in individual CAFs (bottom rows) and cancer cells (top rows) in the scRNA-seq data, using the "lenient" definitions for CAFs. Selected ESGs are labelled at the side of each co-expression matrix.

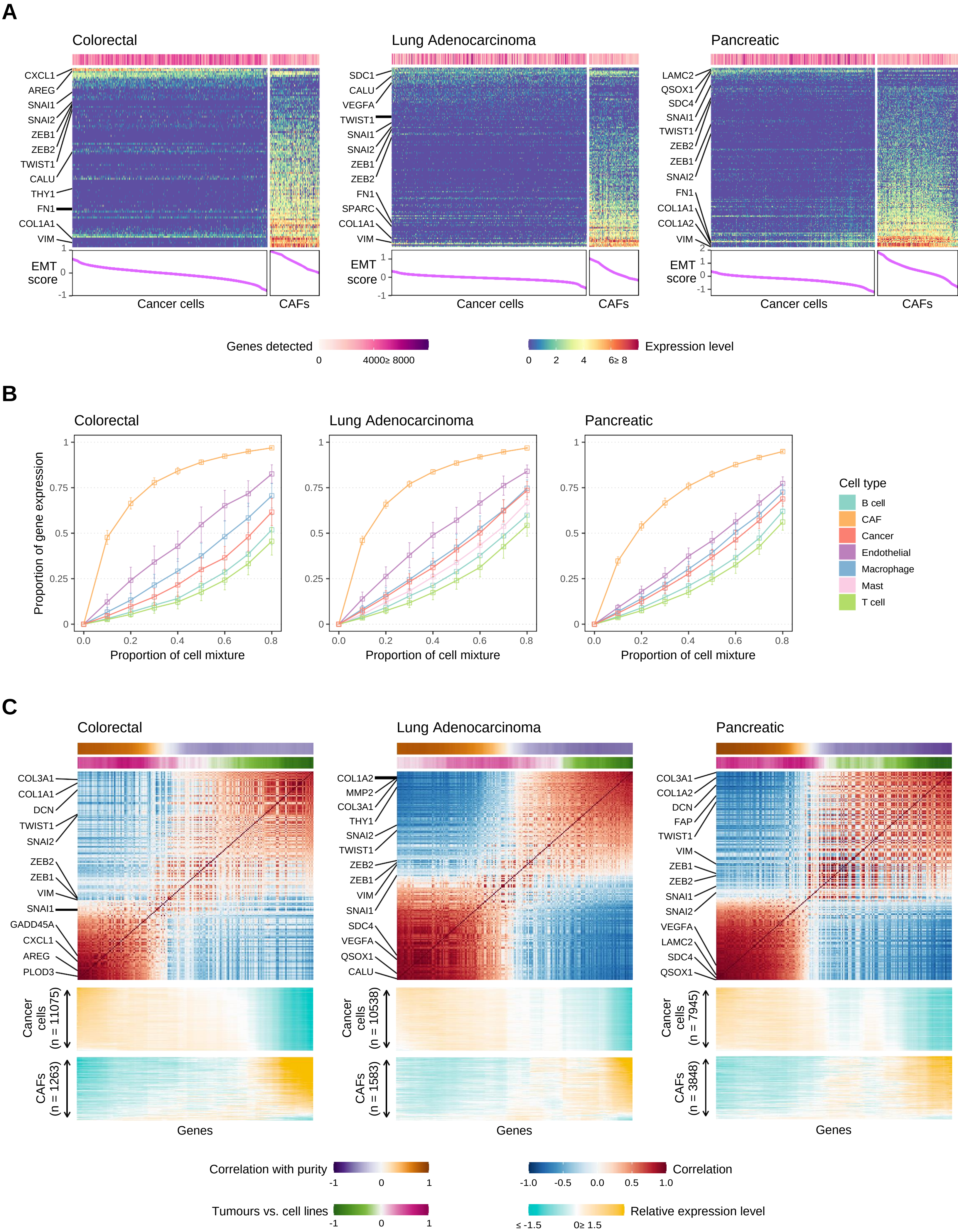
A
Colorectal
Lung Adenocarcinoma
Pancreatic
CXCL1
AREG
SNAI1
SNAI2
ZEB1
ZEB2
TWIST1
CALU
THY1
FN1
COL1A1
VIM
SDC1
CALU
VEGFA
TWIST1
SNAI1
SNAI2
ZEB1
ZEB2
FN1
SPARC
COL1A1
VIM
LAMC2
QSOX1
SDC4
SNAI1
TWIST1
ZEB2
ZEB1
SNAI2
FN1
COL1A1
COL1A2
VIM
EMT score
Cancer cells
CAFs
Genes detected
0
4000
≥ 8000
Expression level
0
2
4
6
≥ 8
B
Colorectal
Lung Adenocarcinoma
Pancreatic
Proportion of gene expression
Proportion of cell mixture
Cell type
B cell
CAF
Cancer
Endothelial
Macrophage
Mast
T cell
C
Colorectal
Lung Adenocarcinoma
Pancreatic
COL3A1
COL1A1
DCN
TWIST1
SNAI2
ZEB2
ZEB1
VIM
SNAI1
GADD45A
CXCL1
AREG
PLOD3
COL1A2
MMP2
COL3A1
THY1
SNAI2
TWIST1
ZEB2
ZEB1
VIM
SNAI1
SDC4
VEGFA
QSOX1
CALU
COL3A1
COL1A2
DCN
FAP
TWIST1
VIM
ZEB1
ZEB2
SNAI1
SNAI2
VEGFA
LAMC2
SDC4
QSOX1
Cancer cells (n = 11075)
CAFs (n = 1263)
Cancer cells (n = 10538)
CAFs (n = 1583)
Cancer cells (n = 7945)
CAFs (n = 3848)
Genes
Correlation with purity
-1
0
1
Correlation
-1.0
-0.5
0.0
0.5
1.0
Tumours vs. cell lines
-1
0
1
Relative expression level
≤ -1.5
0
≥ 1.5

Figure S7. Summary of analysis of scRNA-seq data normalised by scran[8]. (**A**) Heatmaps showing expression levels of ESGs (rows) in cancer cells and CAFs (columns) in 3 scran-normalised scRNA-seq datasets. Columns (cells) are ordered by their EMT scores (see **Methods**), which are shown in the line graphs below the heatmaps. The bar above each heatmap shows the number of genes detected in each cell. (**B**) Line plots showing the relative contributions of different cell types to ESG expression, for various fractions of tumour composition, in simulated bulk expression profiles based on the 3 scran-normalised scRNA-seq datasets shown in (**A**). Each point represents the average proportion of EMT signature gene expression coming from the corresponding cell type in a collection of 100 simulated tumours with the given fraction of that cell type and varying proportions of the other cell types. Error bars show the standard deviation over the set of 100 simulations. (**C**) ESG co-expression matrices derived from simulated bulk expression data based on the 3 scran-normalised scRNA-seq datasets shown in (**A**), ordered by the SPIN side-to-side algorithm[7] with slight modifications (see **Methods**). ESGs are annotated with two colour-coded panels at the top: (1) correlations with simulated tumour purity (Pearson correlation coefficient); and (2) comparison of expression levels in simulated tumours versus in cell lines, where positive numbers indicate higher expression in tumours than in cell lines. Heatmaps below the co-expression matrices show the relative expression levels of ESGs in individual CAFs (bottom rows) and cancer cells (top rows) in the scRNA-seq data. Selected ESGs are labelled at the side of each co-expression matrix.

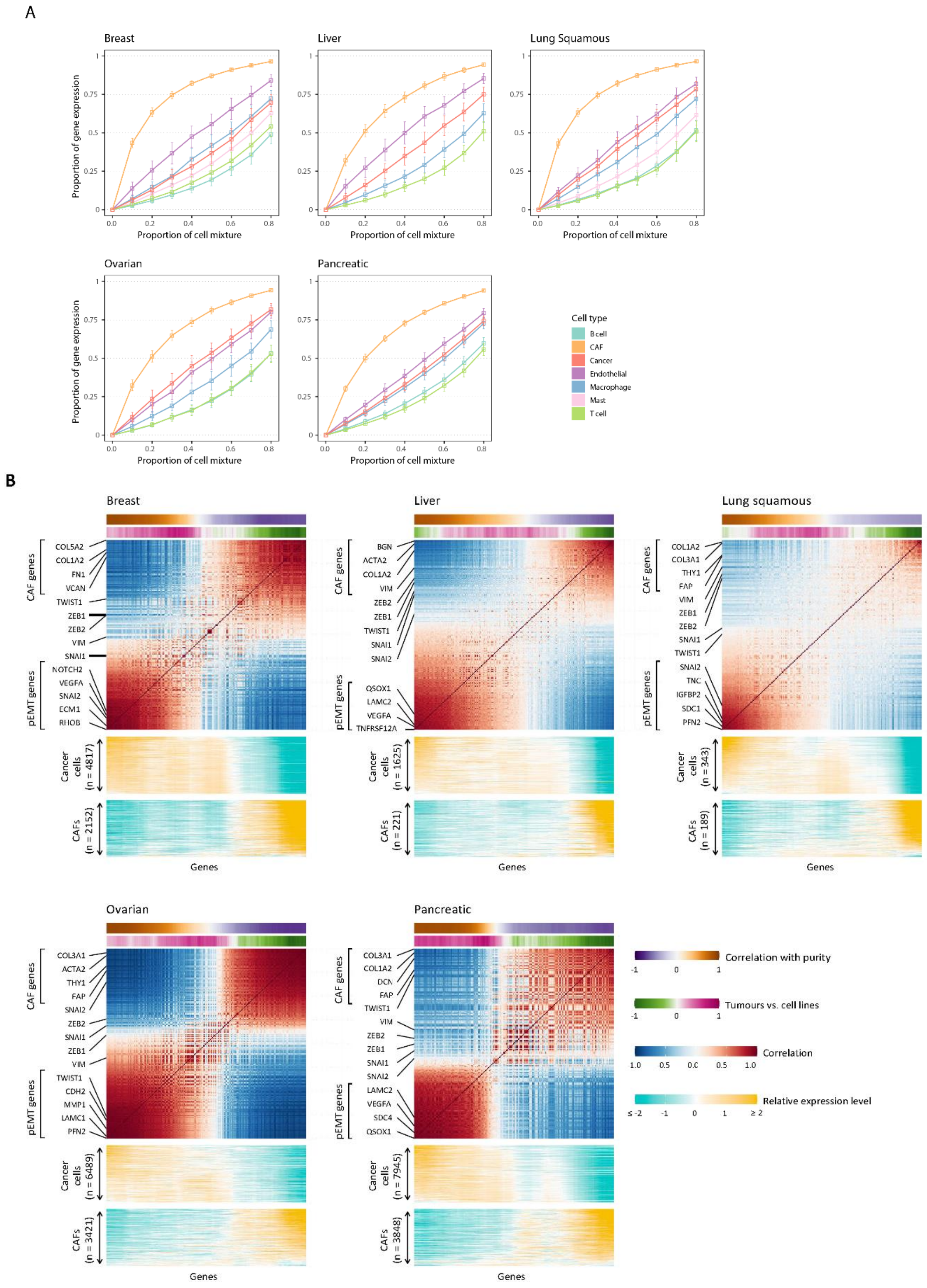

A
Breast
Liver
Lung Squamous
Ovarian
Pancreatic
Proportion of gene expression
Proportion of cell mixture
Cell type
B cell
CAF
Cancer
Endothelial
Macrophage
Mast
T cell
B
Breast
Liver
Lung squamous
Ovarian
Pancreatic
CAF genes
pEMT genes
COL5A2
COL1A2
FN1
VCAN
TWIST1
ZEB1
ZEB2
VIM
SNAI1
NOTCH2
VEGFA
SNAI2
ECM1
RHOB
BGN
ACTA2
QSOX1
LAMC2
TNFRSF12A
COL3A1
THY1
FAP
TNC
IGFBP2
SDC1
PFN2
CDH2
MMP1
DCN
SDC4
Cancer cells (n = 4817)
CAFs (n = 2152)
Cancer cells (n = 1625)
CAFs (n = 221)
Cancer cells (n = 343)
CAFs (n = 189)
Cancer cells (n = 6489)
CAFs (n = 3421)
Cancer cells (n = 7945)
CAFs (n = 3848)
Genes
Correlation with purity
Tumours vs. cell lines
Correlation
Relative expression level

Figure S8. Contribution of different cell types to expression of ESGs in simulated bulk tumours. (**A**) Line plots showing the relative contributions of different cell types to ESG expression (EMT signal), for various fractions of tumour composition, in simulated bulk expression profiles based on the 5 scRNA-seq datasets not shown in **Fig. 2A**. Each point represents the average proportion of EMT signature gene expression coming from the corresponding cell type in a collection of 100 simulated tumours with the given fraction of that cell type and varying proportions of the other cell types. Error bars show the standard deviation over the set of 100 simulations. (**B**) ESG co-expression matrices derived from simulated bulk expression profiles based on the 5 scRNA-seq datasets not shown in **Fig. 2B**, ordered by the SPIN side-to-side algorithm[7] with slight modifications (see **Methods**). ESGs are annotated with two colour-coded panels at the top: (1) correlations with simulated tumour purity (Pearson correlation coefficient); and (2) comparison of expression levels in simulated tumours versus in cell lines, where positive numbers indicate higher expression in tumours than in cell lines, and vice versa. Heatmaps below the co-expression matrices show the relative expression levels of ESGs in individual CAFs and cancer cells in the scRNA-seq data. Selected ESGs are labelled at the side of each co-expression matrix. Source data are provided as a Source Data file.

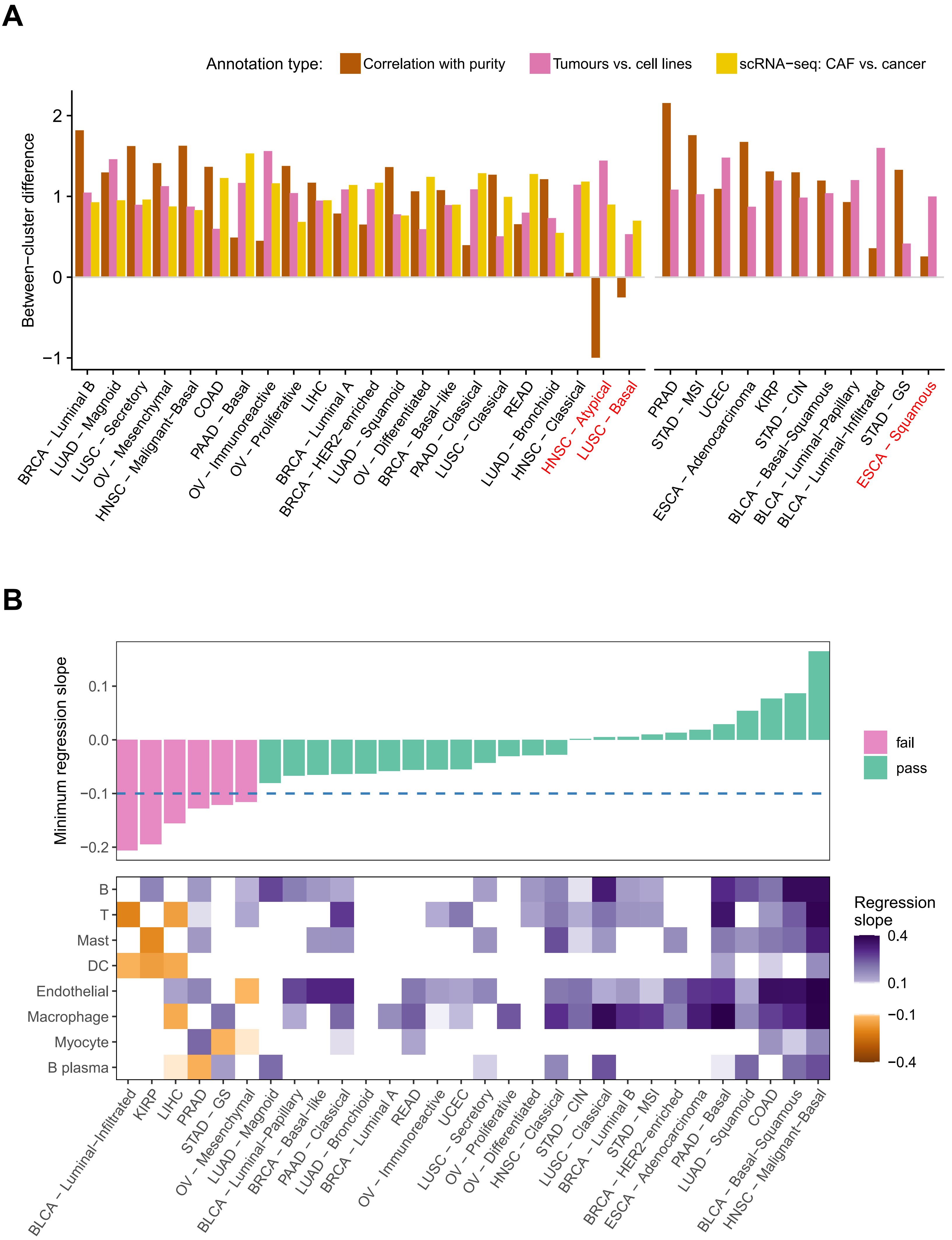
A
Annotation type:
Correlation with purity
Tumours vs. cell lines
scRNA−seq: CAF vs. cancer
Between−cluster difference
2
1
0
−1
BRCA – Luminal B
LUAD – Magnoid
LUSC – Secretory
OV – Mesenchymal
HNSC – Malignant-Basal
COAD
PAAD – Basal
OV – Immunoreactive
OV – Proliferative
LIHC
BRCA – Luminal A
BRCA – HER2-enriched
LUAD – Squamoid
OV – Differentiated
BRCA – Basal-like
PAAD – Classical
LUSC – Classical
READ
LUAD – Bronchioid
HNSC – Classical
HNSC – Atypical
LUSC – Basal
PRAD
STAD – MSI
UCEC
ESCA – Adenocarcinoma
KIRP
STAD – CIN
BLCA – Basal-Squamous
BLCA – Luminal-Papillary
BLCA – Luminal-Infiltrated
STAD – GS
ESCA – Squamous
B
Minimum regression slope
0.1
0.0
−0.1
−0.2
fail
pass
B
T
Mast
DC
Endothelial
Macrophage
Myocyte
B plasma
Regression slope
0.4
0.1
−0.1
−0.4
BLCA – Luminal-Infiltrated
KIRP
LIHC
PRAD
STAD – GS
OV – Mesenchymal
LUAD – Magnoid
BLCA – Luminal-Papillary
BRCA – Basal-like
PAAD – Classical
LUAD – Bronchioid
BRCA – Luminal A
READ
OV – Immunoreactive
UCEC
LUSC – Secretory
OV – Proliferative
OV – Differentiated
HNSC – Classical
STAD – CIN
LUSC – Classical
BRCA – Luminal B
STAD – MSI
BRCA – HER2-enriched
ESCA – Adenocarcinoma
PAAD – Basal
LUAD – Squamoid
COAD
BLCA – Basal-Squamous
HNSC – Malignant-Basal

Figure S9. Quality control for the TCGA deconvolution results. (**A**) Bar plot of scores for each cancer type/subtype and each of the three validation measures used to annotate the pEMT and CAF clusters in the ordered ESG co-expression matrix for that cancer type/subtype. The score for a given measure and cancer type/subtype represents the average between-cluster difference in values for that measure, reflecting its strength of agreement with the separation of the ESGs into pEMT and CAF clusters. The cancer types are ordered by the number of validation measures present, then by the average of the scores for those measures. HNSC Atypical, LUSC Basal and ESCA Squamous were excluded from further analysis due to weak combined evidence from their validation measures. (**B**) Lower panel: heatmap showing, for each cell type (row) and for each cancer type not excluded following the analysis in (**A**) (column), the slope of a linear regression model quantifying the change in correlation between ESGs and marker genes for that cell type along the ordered ESG list. Upper panel: bar plot showing the minimum such regression slope for each cancer type/subtype, with the horizontal dashed line indicating the cut-off of -0.1. Cancer types/subtypes for which the minimum regression slope was less than -0.1 were excluded from further analysis.

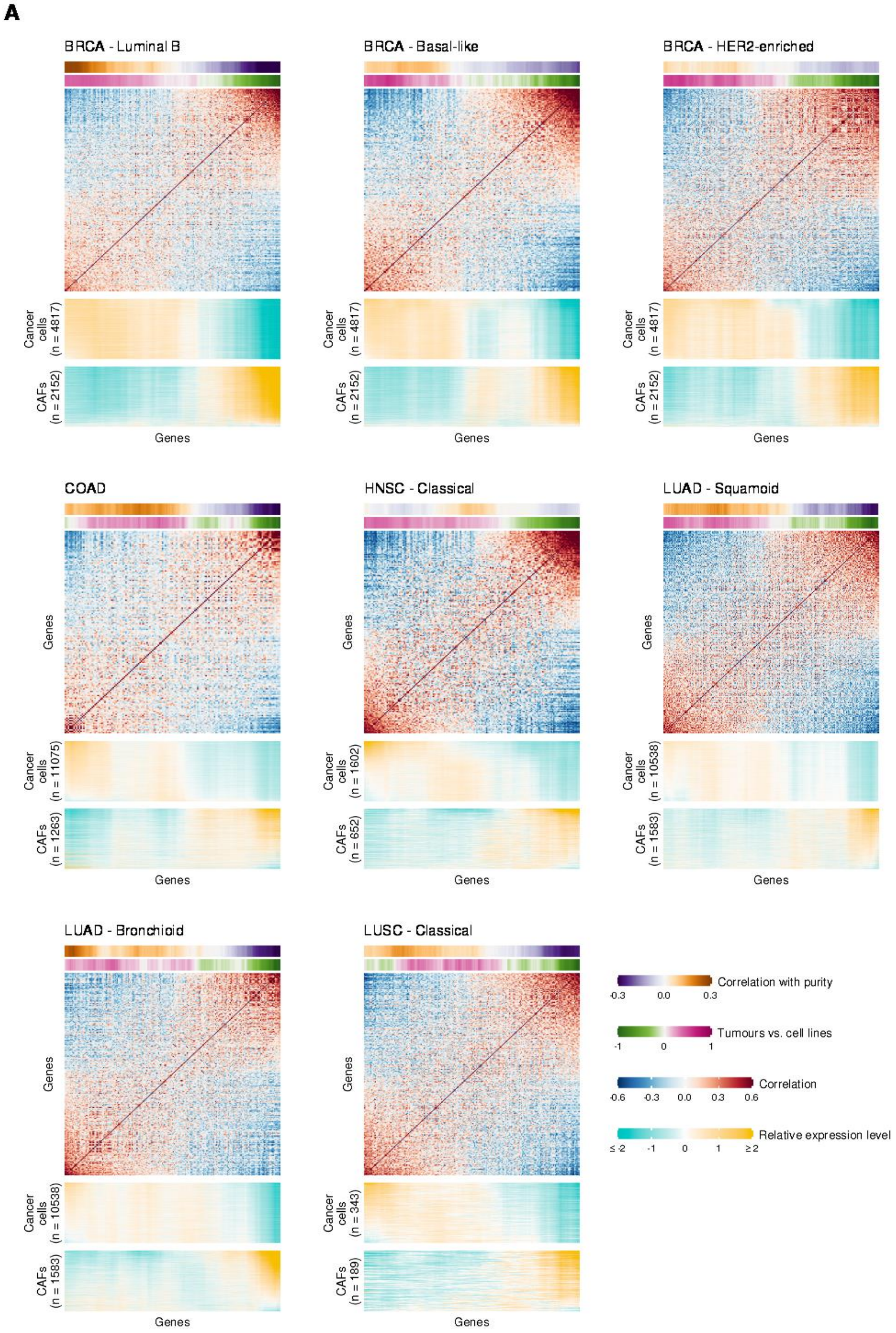

A
BRCA - Luminal B
BRCA - Basal-like
BRCA - HER2-enriched
Cancer cells (n = 4817)
CAFs (n = 2152)
Genes
COAD
HNSC - Classical
LUAD - Squamoid
Cancer cells (n = 11075)
CAFs (n = 1263)
Cancer cells (n = 1602)
CAFs (n = 652)
Cancer cells (n = 10538)
CAFs (n = 1583)
LUAD - Bronchioid
LUSC - Classical
Cancer cells (n = 343)
CAFs (n = 189)
Correlation with purity
-0.3 0.0 0.3
Tumours vs. cell lines
-1 0 1
Correlation
-0.6 -0.3 0.0 0.3 0.6
Relative expression level
≤ -2 -1 0 1 ≥ 2

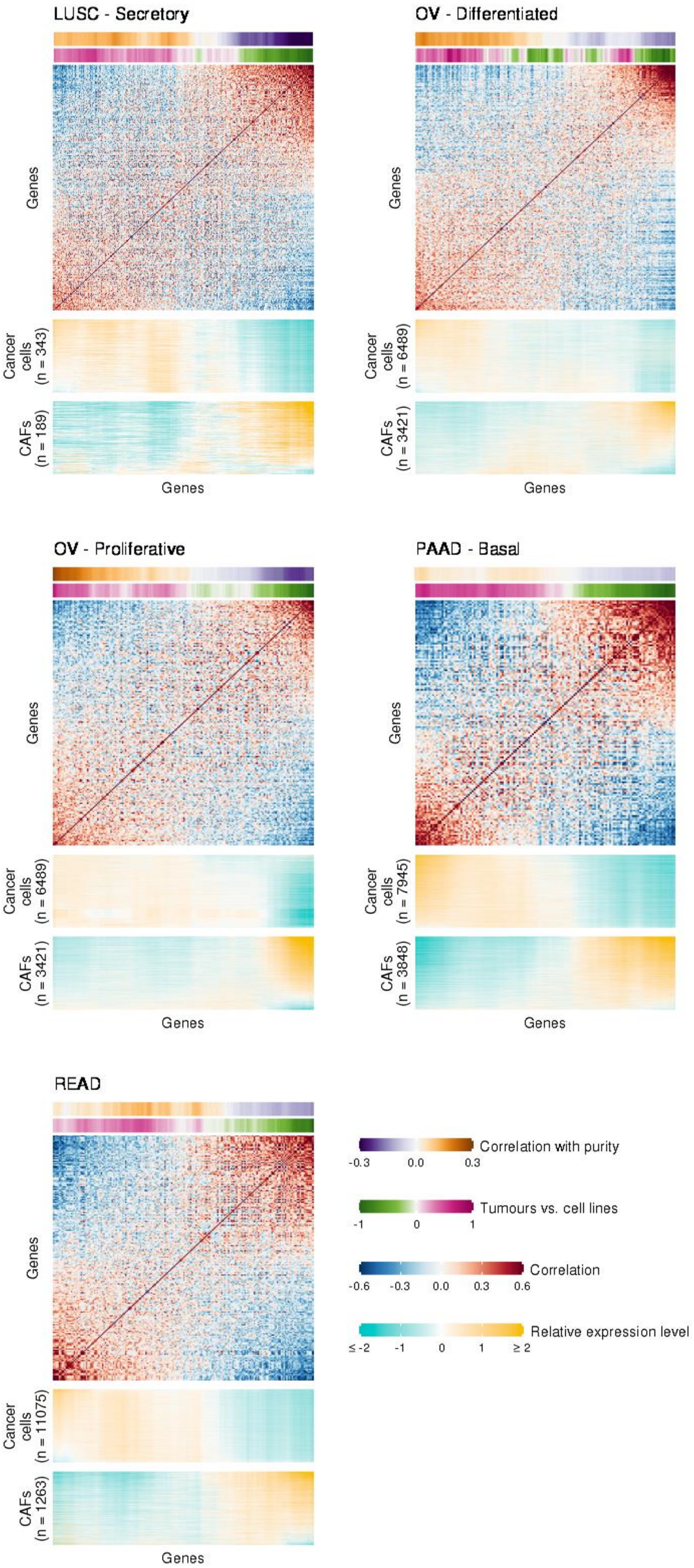
LUSC - Secretory
OV - Differentiated
OV - Immunoreactive
OV - Proliferative
PAAD - Basal
PAAD - Classical
READ
Genes
Cancer cells (n = 343)
CAFs (n = 189)
Cancer cells (n = 6489)
CAFs (n = 3421)
Cancer cells (n = 7945)
CAFs (n = 3848)
Cancer cells (n = 11075)
CAFs (n = 1263)
Correlation with purity
-0.3 0.0 0.3
Tumours vs. cell lines
-1 0 1
Correlation
-0.6 -0.3 0.0 0.3 0.6
Relative expression level
≤ -2 -1 0 1 ≥ 2

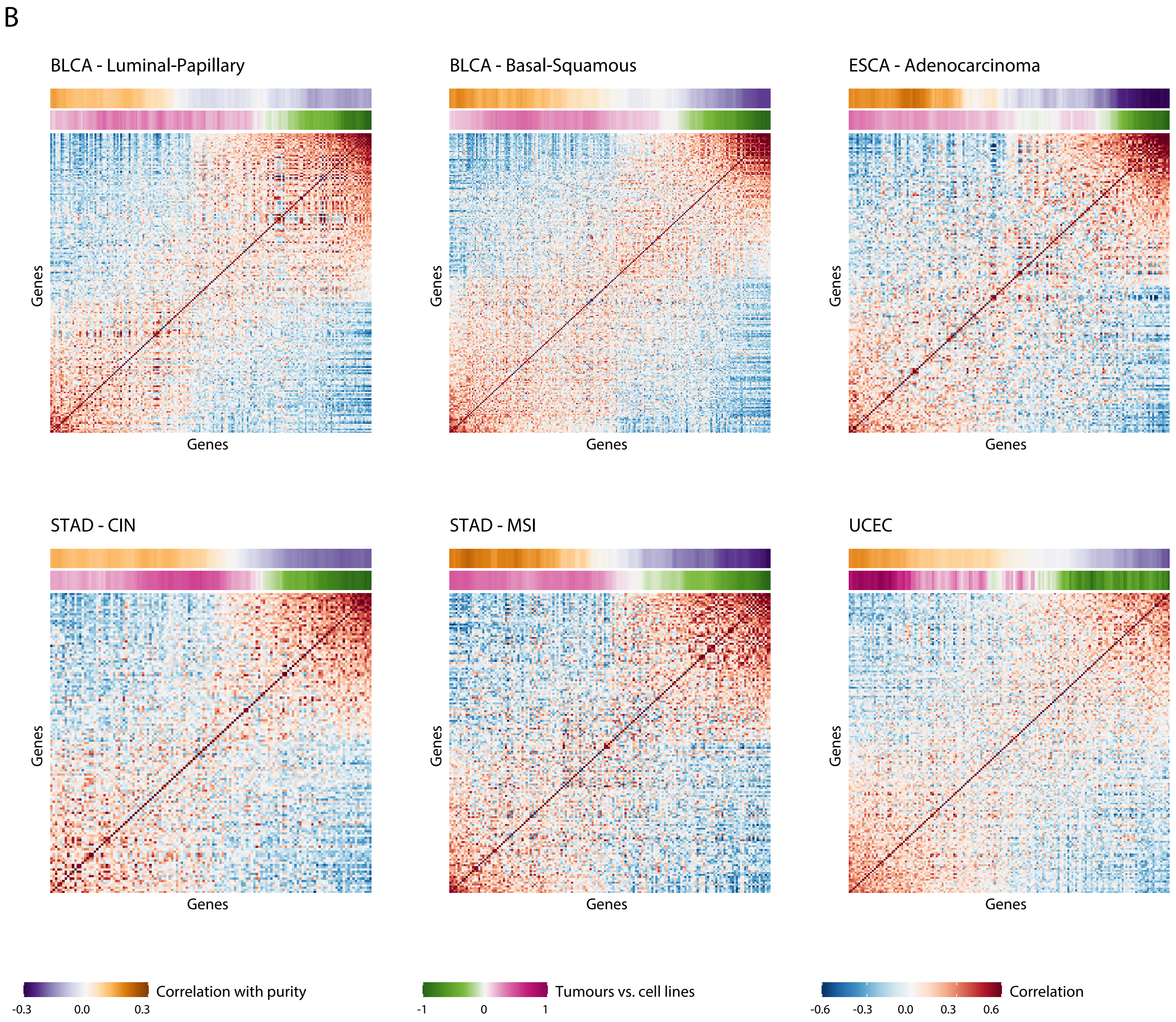
B
BLCA - Luminal-Papillary
BLCA - Basal-Squamous
ESCA - Adenocarcinoma
STAD - CIN
STAD - MSI
UCEC
Genes
Correlation with purity
-0.3 0.0 0.3
Tumours vs. cell lines
-1 0 1
Correlation
-0.6 -0.3 0.0 0.3 0.6

Figure S10. Deconvolution of cancer and CAF ESG expression from TCGA bulk expression profiles. (**A**) ESG co-expression matrices derived from TCGA bulk expression data for the 15 cancer types/subtypes which have accompanying scRNA-seq data and passed the quality control criteria but which were not shown in **Fig. 3A**. Each matrix is ordered by the SPIN side-to-side algorithm[7] with slight modifications (see **Methods**) and annotated by two colour-coded panels at the top showing (1) Pearson correlations with estimates of tumour purity computed by ABSOLUTE[9]; and (2) comparison of expression levels in tumours versus in cell lines, where positive numbers indicate higher expression in tumours than in cell lines. Heatmaps below the co-expression matrices show the relative expression levels of ESGs in individual CAFs and cancer cells in the relevant scRNA-seq dataset. (**B**) ESG co-expression matrices as in (**A**) for the 6 cancer types which passed the quality control criteria but which do not have accompanying scRNA-seq data. Source data are provided as a Source Data file.

Common EMT and stroma genes

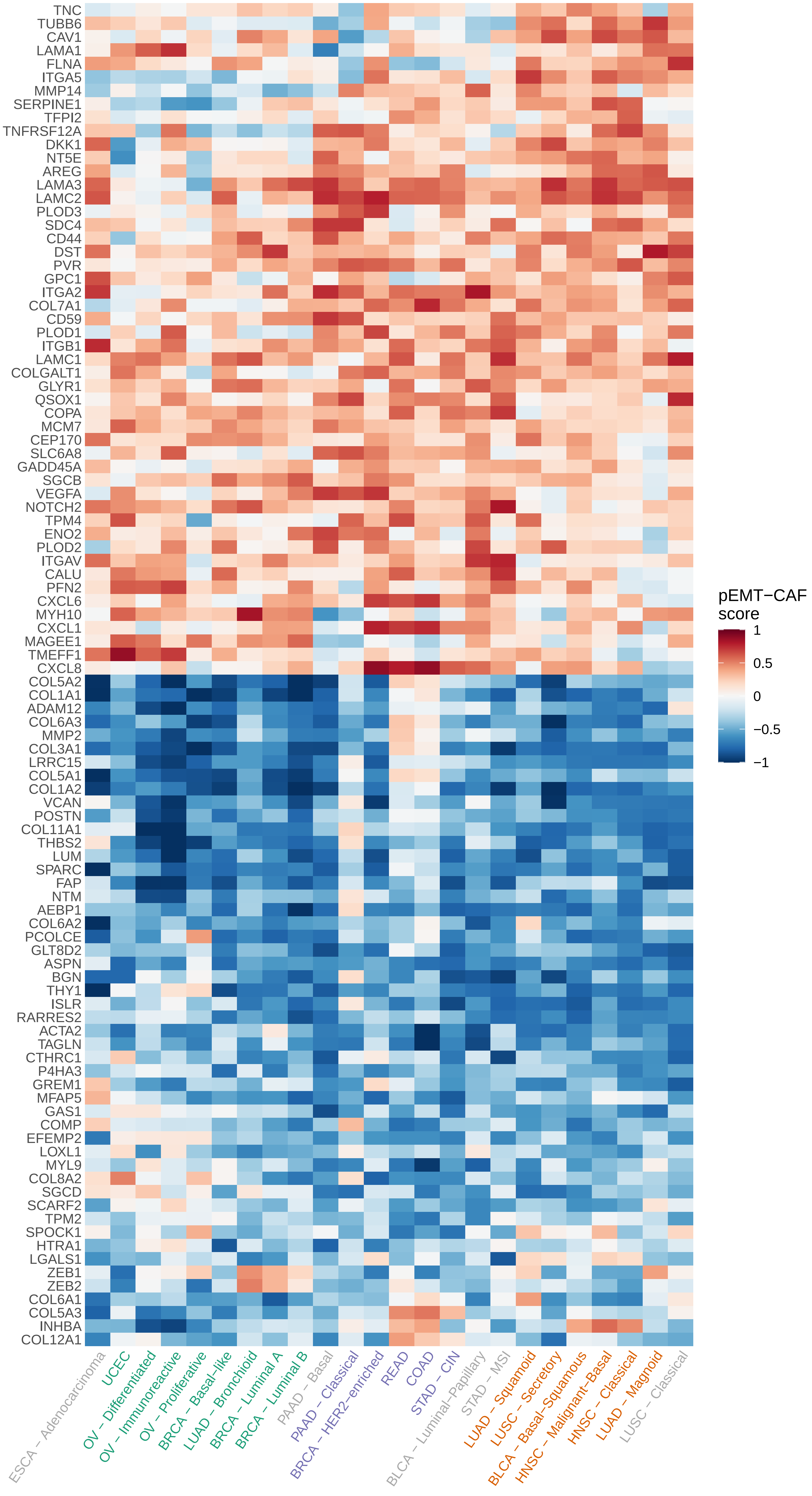

Figure S11. Summary of ESG association with cancer cells and CAFs across cancer types/subtypes. Heatmap showing the pEMT-CAF scores for a set of 100 ESGs (rows) comprising the 50 genes most commonly occurring in the pEMT and CAF clusters across all those cancer types/subtypes (columns) whose TCGA deconvolution results passed quality control. ESGs are ordered by the SPIN Neighborhood algorithm[7], and the cancer types are ordered by hierarchical clustering of pairwise correlations between their pEMT-CAF scores, and coloured by their pEMT cluster assignments. Source data are provided as a Source Data file.

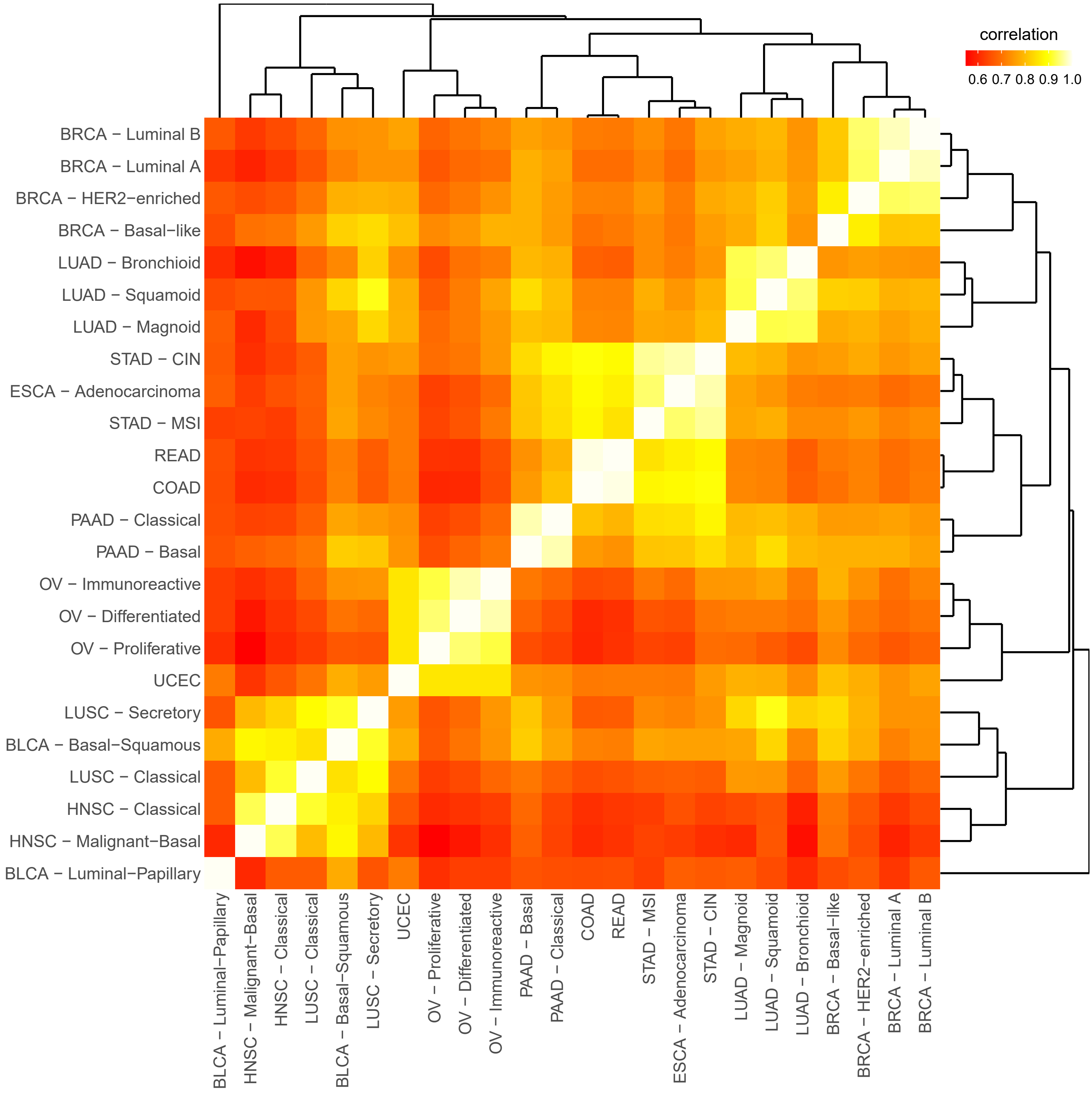

correlation
0.6 0.7 0.8 0.9 1.0
BRCA − Luminal B
BRCA − Luminal A
BRCA − HER2−enriched
BRCA − Basal−like
LUAD − Bronchioid
LUAD − Squamoid
LUAD − Magnoid
STAD − CIN
ESCA − Adenocarcinoma
STAD − MSI
READ
COAD
PAAD − Classical
PAAD − Basal
OV − Immunoreactive
OV − Differentiated
OV − Proliferative
UCEC
LUSC − Secretory
BLCA − Basal−Squamous
LUSC − Classical
HNSC − Classical
HNSC − Malignant−Basal
BLCA − Luminal−Papillary

Figure S12. Global similarity between cancer types/subtypes. Heatmap showing the similarities between cancer types/subtypes measured by average correlation between average expression levels of the most highly variable genes. Both axes of the heatmap are ordered by hierarchical clustering with average linkage.

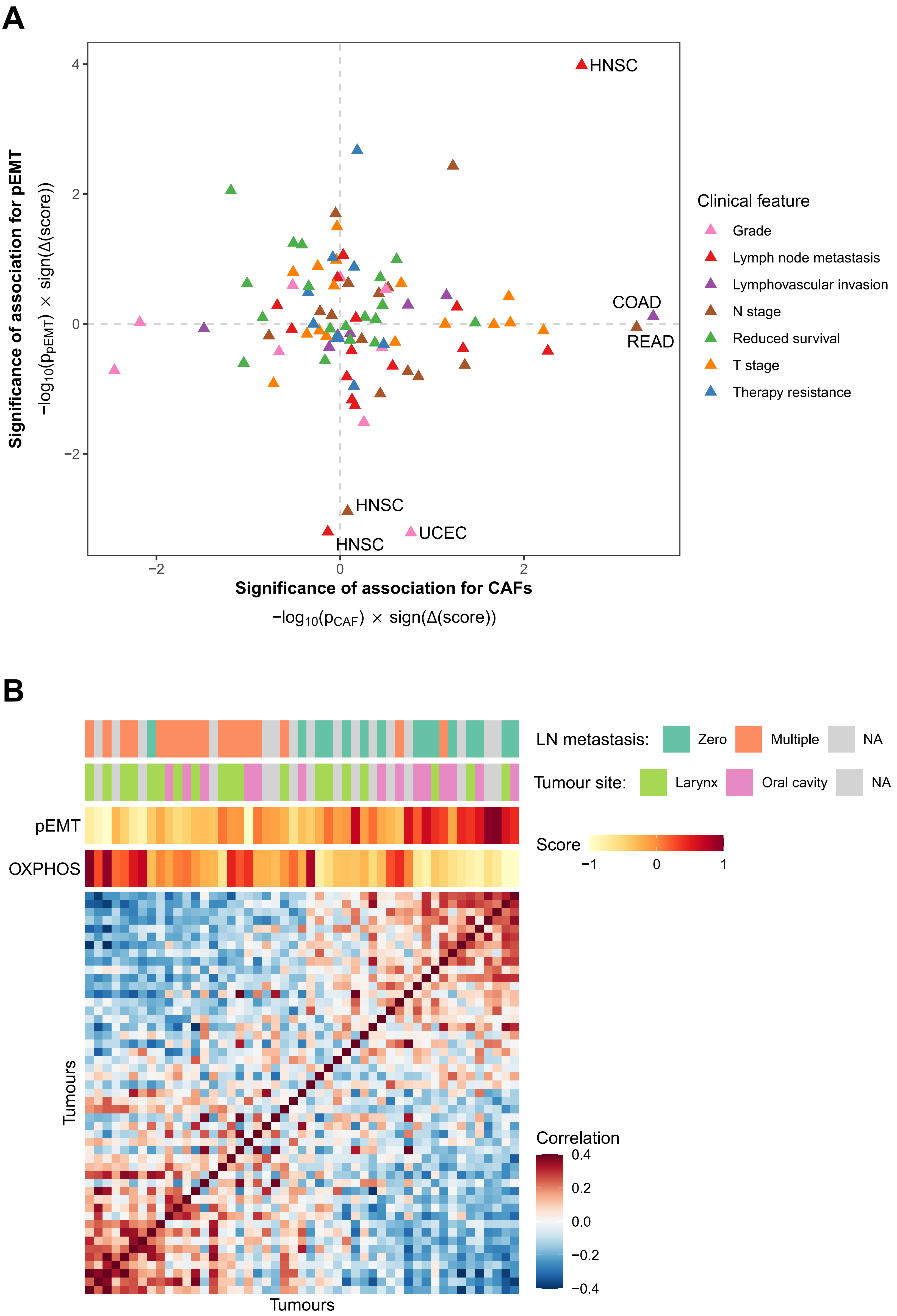

A
HNSC
COAD
READ
HNSC
UCEC
HNSC
Significance of association for pEMT
−log10(ppEMT) × sign(Δ(score))
Significance of association for CAFs
−log10(pCAF) × sign(Δ(score))
Clinical feature
Grade
Lymph node metastasis
Lymphovascular invasion
N stage
Reduced survival
T stage
Therapy resistance
B
LN metastasis:
Zero
Multiple
NA
Tumour site:
Larynx
Oral cavity
NA
pEMT
OXPHOS
Score
Tumours
Tumours
Correlation

Figure S13. Association of pEMT and CAF signatures with clinical features. (**A**) Scatterplots showing the significance (quantified as –log10(p-value) based on a two-sided Wilcoxon rank-sum test, without adjustment for multiple comparisons) of the association of signatures for pEMT (Y-axis) and CAFs (X-axis) with seven clinical features (indicated by colour) reflecting worse prognosis. Points are labelled with their corresponding cancer types if they pass an adjusted significance threshold corresponding to an FDR of 0.05. Source data are provided as a Source Data file. (**B**) Correlation matrix of HNSC Classical tumours in TCGA data, ordered by the SPIN side-to-side algorithm[7] and annotated with coloured bars at the top showing lymph node (LN) metastasis, tumour site and scores for pEMT and oxidative phosphorylation (OXPHOS) gene signatures.

Supplementary References

1. Qian, J. *et al.* A pan-cancer blueprint of the heterogeneous tumor microenvironment revealed by single-cell profiling. *Cell Res.* **30**, 745–762 (2020).
2. Lee, H.-O. *et al.* Lineage-dependent gene expression programs influence the immune landscape of colorectal cancer. *Nat. Genet.* 1–10 (2020) doi:10.1038/s41588-020-0636-z.
3. Puram, S. V. *et al.* Single-Cell Transcriptomic Analysis of Primary and Metastatic Tumor Ecosystems in Head and Neck Cancer. *Cell* **171**, 1611-1624.e24 (2017).
4. Ma, L. *et al.* Tumor Cell Biodiversity Drives Microenvironmental Reprogramming in Liver Cancer. *Cancer Cell* **36**, 418-430.e6 (2019).
5. Kim, N. *et al.* Single-cell RNA sequencing demonstrates the molecular and cellular reprogramming of metastatic lung adenocarcinoma. *Nat. Commun.* **11**, 2285 (2020).
6. Peng, J. *et al.* Single-cell RNA-seq highlights intra-tumoral heterogeneity and malignant progression in pancreatic ductal adenocarcinoma. *Cell Res.* **29**, 725–738 (2019).
7. Tsafrir, D. *et al.* Sorting points into neighborhoods (SPIN): data analysis and visualization by ordering distance matrices. *Bioinformatics* **21**, 2301–2308 (2005).
8. L. Lun, A. T., Bach, K. & Marioni, J. C. Pooling across cells to normalize single-cell RNA sequencing data with many zero counts. *Genome Biol.* **17**, 75 (2016).
9. Carter, S. L. *et al.* Absolute quantification of somatic DNA alterations in human cancer. *Nat. Biotechnol.* **30**, 413–421 (2012).
