# Supplementary material for: Decoupling epithelial-mesenchymal transitions from stromal profiles by integrative expression analysis
Source: Nat Commun. 2021 May 10;12:2592. doi: 10.1038/s41467-021-22800-1 (PMC8110844; doi:10.1038/s41467-021-22800-1)
Supplement: Supplementary file 2 — Reporting Summary [file 41467_2021_22800_MOESM2_ESM.pdf]

nature research

Corresponding author(s): Itay Tirosh

Last updated by author(s): 25/02/2021

Reporting Summary

Nature Research wishes to improve the reproducibility of the work that we publish. This form provides structure for consistency and transparency in reporting. For further information on Nature Research policies, see our Editorial Policies and the Editorial Policy Checklist.

Statistics

For all statistical analyses, confirm that the following items are present in the figure legend, table legend, main text, or Methods section.

n/a	Confirmed	
☒	☐	The exact sample size (*n*) for each experimental group/condition, given as a discrete number and unit of measurement
☐	☒	A statement on whether measurements were taken from distinct samples or whether the same sample was measured repeatedly
☐	☒	The statistical test(s) used AND whether they are one- or two-sided<br>*Only common tests should be described solely by name; describe more complex techniques in the Methods section.*
☐	☒	A description of all covariates tested
☐	☒	A description of any assumptions or corrections, such as tests of normality and adjustment for multiple comparisons
☐	☒	A full description of the statistical parameters including central tendency (e.g. means) or other basic estimates (e.g. regression coefficient) AND variation (e.g. standard deviation) or associated estimates of uncertainty (e.g. confidence intervals)
☒	☐	For null hypothesis testing, the test statistic (e.g. *F*, *t*, *r*) with confidence intervals, effect sizes, degrees of freedom and *P* value noted<br>*Give P values as exact values whenever suitable.*
☒	☐	For Bayesian analysis, information on the choice of priors and Markov chain Monte Carlo settings
☒	☐	For hierarchical and complex designs, identification of the appropriate level for tests and full reporting of outcomes
☐	☒	Estimates of effect sizes (e.g. Cohen's *d*, Pearson's *r*), indicating how they were calculated

Our web collection on statistics for biologists contains articles on many of the points above.

Software and code

Policy information about availability of computer code

Data collection	All data used in this study was downloaded manually and processed using R version 3.6.3.
Data analysis	All data analysis in this study was performed using R version 3.6.3.

For manuscripts utilizing custom algorithms or software that are central to the research but not yet described in published literature, software must be made available to editors and reviewers. We strongly encourage code deposition in a community repository (e.g. GitHub). See the Nature Research guidelines for submitting code & software for further information.

Data

Policy information about availability of data

All manuscripts must include a data availability statement. This statement should provide the following information, where applicable:

- Accession codes, unique identifiers, or web links for publicly available datasets
- A list of figures that have associated raw data
- A description of any restrictions on data availability

This study involved re-analysis of published datasets, including scRNA-seq datasets available through the original studies, as described in Table S1, and bulk tumor datasets from TCGA, available at http://gdac.broadinstitute.org/.
The breast and ovarian cancer datasets of Qian et al. are available in the ArrayExpress database under accession code E-MTAB-8107.
The colorectal cancer dataset of Lee et al. is available in the NCBI Gene Expression Omnibus (GEO) database under accession code GSE132465.
The head and neck cancer (HNSCC) dataset of Puram et al. is available in the GEO database under accession code GSE103322.
The liver cancer dataset of Ma et al. is available in the GEO database under accession code GSE125449.
The lung adenocarcinoma dataset of Kim et al. is available in the GEO database under accession code GSE131907.
The lung cancer dataset of Qian et al. is available in the ArrayExpress database under accession codes E-MTAB-6149 and E-MTAB-6653.

The pancreatic cancer dataset of Peng et al. is available in the Genome Sequence Archive database under accession code CRA001160.
The TCGA datasets were downloaded from http://gdac.broadinstitute.org/. For each cancer type, the corresponding bulk RNA-seq dataset was downloaded by selecting the 'illuminahiseq_rnaseqv2-RSEM_genes' link, and the clinical dataset by selecting 'Clinical_Pick_Tier1'.
The MSigDB Hallmark EMT gene set is available on the GSEA-MSigDB website.
The remaining data are available from the authors upon request.

Field-specific reporting

Please select the one below that is the best fit for your research. If you are not sure, read the appropriate sections before making your selection.

☒ Life sciences ☐ Behavioural & social sciences ☐ Ecological, evolutionary & environmental sciences

For a reference copy of the document with all sections, see nature.com/documents/nr-reporting-summary-flat.pdf

Life sciences study design

All studies must disclose on these points even when the disclosure is negative.

Sample size	Sample sizes were determined by the publicly available data. For the scRNA-seq analysis, we considered scRNA-seq datasets for epithelial cancer types. Where there were multiple datasets available for a given cancer type, we selected the dataset having the highest number of samples in which cancer cells could be clearly distinguished from nonmalignant cells (by inferred copy number alterations), and which, after this separation, contained at least 100 cancer cells. Eight such datasets were analysed, which was sufficient to demonstrate the consistently low mesenchymal gene expression in cancer cells relative to CAFs and to test our deconvolution method, but it is not exhaustive and may miss rare mesenchymal subpopulations, as discussed in the article. TCGA bulk RNA-seq datasets were also considered for all epithelial cancer types, but cancer types were excluded if their datasets comprised fewer than 100 tumours. Individual subtypes within a dataset were excluded if they comprised 30 or fewer tumours. These numbers were chosen so that the collection of tumours for a given cancer types adequately represented the diversity both within and between subtypes, and in particular, the diversity in mesenchymal gene expression. The number of datasets included, and the sample sizes within each, were sufficient to detect a division of pEMT profiles into three clusters, and to identify statistically significant associations with clinical features that remained significant after adjustment for multiple comparisons.
Data exclusions	Certain samples or datasets were excluded either based on criteria established in literature or due to sample sizes which we judged insufficient. For the HNSCC dataset of Puram et al., only 10 of the 18 samples were considered, since these were the ones analysed in the original study (having the most cancer cells). Where multiple scRNA-seq datasets existed for a given cancer type, we selected datasets having the highest number of samples in which cancer cells could be clearly distinguished from nonmalignant cells (by inferred copy number alterations), and which, after this separation, contained at least 100 cancer cells. Other datasets for this cancer type were excluded, as they contained fewer usable samples. TCGA bulk RNA-seq datasets were excluded if they comprised fewer than 100 tumours, while individual subtypes were excluded if they comprised 30 or fewer tumours. This was to ensure that the data for each cancer type/subtype adequately represented the diversity both within and between subtypes.
Replication	Much of our analysis was fully deterministic. Where there was an element of randomness, such as when applying the SPIN algorithm or when sampling cells, reproducibility was ensured by setting a seed, such that the same result would be obtained each time the code was run. When conducting simulations of bulk expression profiles by sampling and aggregating profiles of single cells, we chose a sample number high enough to ensure stability of the result (1000 for each cancer type when applying the deconvolution method to simulated bulk profiles; 100 per data point when measuring the contributions of different cell types to the mesenchymal signal). When applying the deconvolution method to TCGA bulk expression data, we tested multiple parameter settings for each cancer type, and selected parameters around which the results were stable.
Randomization	This is not relevant to our study, as the same analysis was applied to all datasets, and they were not divided into groups.
Blinding	This is not relevant to our study, as the same analysis was applied to all datasets, and they were not divided into groups.

Reporting for specific materials, systems and methods

We require information from authors about some types of materials, experimental systems and methods used in many studies. Here, indicate whether each material, system or method listed is relevant to your study. If you are not sure if a list item applies to your research, read the appropriate section before selecting a response.

Materials & experimental systems

n/a	Involved in the study
☒	☐ Antibodies
☒	☐ Eukaryotic cell lines
☒	☐ Palaeontology and archaeology
☒	☐ Animals and other organisms
☒	☐ Human research participants
☒	☐ Clinical data
☒	☐ Dual use research of concern

Methods

n/a	Involved in the study
☒	☐ ChIP-seq
☒	☐ Flow cytometry
☒	☐ MRI-based neuroimaging
